# Filamentous temperature sensitive mutant Z: a putative target to combat antibacterial resistance

**DOI:** 10.1039/d3ra00013c

**Published:** 2023-04-11

**Authors:** Sumaiya Kifayat, Vidyasrilekha Yele, Akram Ashames, Dilep Kumar Sigalapalli, Richie R. Bhandare, Afzal B. Shaik, Venkatarathnam Nasipireddy, Bharat Kumar Reddy Sanapalli

**Affiliations:** a Department of Pharmacology, NIMS Institute of Pharmacy, NIMS University Rajasthan Jaipur 303121 India kifayatsumaiya@gmail.com bharathsanapalli@yahoo.in +91-9291661992; b Department of Pharmaceutical Chemistry, NIMS Institute of Pharmacy, NIMS University Rajasthan Jaipur 303121 India vidyasrilekha16@gmail.com; c College of Pharmacy & Health Sciences, Ajman University PO Box 340 Ajman United Arab Emirates; d Center of Medical and Bio-allied Health Sciences Research, Ajman University PO Box 340 Ajman United Arab Emirates r.bhandareh@ajman.ac.ae a.ashames@ajman.ac.ae +97167056240; e Department of Pharmaceutical Chemistry, Vignan Pharmacy College, Jawaharlal Nehru Technological University Vadlamudi 522213 Andhra Pradesh India dileepsigalapalli@gmail.com; f St. Mary's College of Pharmacy, St. Mary's Group of Institutions Guntur, Affiliated to Jawaharlal Nehru Technological University Kakinada Chebrolu Guntur 522212 Andhra Pradesh India bashafoye@gmail.com; g Himalayan Garhwal University Uttarakhand 246169 India vrathnam.nasipireddy@gmail.com

## Abstract

In the pre-antibiotic era, common bacterial infections accounted for high mortality and morbidity. Moreover, the discovery of penicillin in 1928 marked the beginning of an antibiotic revolution, and this antibiotic era witnessed the discovery of many novel antibiotics, a golden era. However, the misuse or overuse of these antibiotics, natural resistance that existed even before the antibiotics were discovered, genetic variations in bacteria, natural selection, and acquisition of resistance from one species to another consistently increased the resistance to the existing antibacterial targets. Antibacterial resistance (ABR) is now becoming an ever-increasing concern jeopardizing global health. Henceforth, there is an urgent unmet need to discover novel compounds to combat ABR, which act through untapped pathways/mechanisms. Filamentous Temperature Sensitive mutant Z (FtsZ) is one such unique target, a tubulin homolog involved in developing a cytoskeletal framework for the cytokinetic ring. Additionally, its pivotal role in bacterial cell division and the lack of homologous structural protein in mammals makes it a potential antibacterial target for developing novel molecules. Approximately 2176 X-crystal structures of FtsZ were available, which initiated the research efforts to develop novel antibacterial agents. The literature has reported several natural, semisynthetic, peptides, and synthetic molecules as FtsZ inhibitors. This review provides valuable insights into the basic crystal structure of FtsZ, its inhibitors, and their inhibitory activities. This review also describes the available *in vitro* detection and quantification methods of FtsZ-drug complexes and the various approaches for determining drugs targeting FtsZ polymerization.

## Abstract 2

Different inhibitors (natural, synthetic and peptides) against FtsZ alongwith detection techniques to quantify the FtsZ-drug complex.

## Graphical abstract

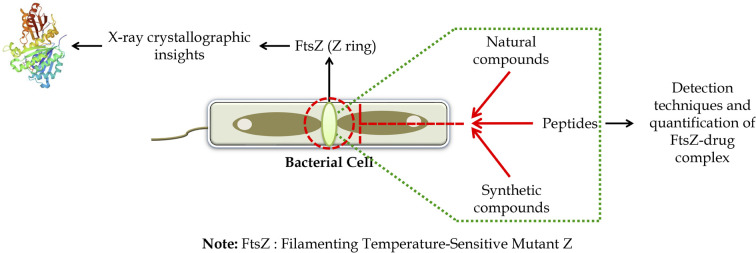

## 1. Introduction

The continuous emergence and rapid spread of antibacterial resistance (ABR) have increased the necessity to discover novel and alternative antibacterial agents that are less susceptible to ABR. However, as a consequence of employing new drugs selected for resistant species, advancing advanced influxes of resistance have confronted ages of alternative antibiotics. The development of new antibiotics in the twentieth century depended on modifying the synthetic structure of pre-existing antibiotics.^1^ Another approach for reducing ABR is the introduction of structurally novel classes of antibiotics that act on therapeutically approved targets. Although many anti-bacterial agents are available in the market, only a small number of them target specific bacterial biological processes, such as the synthesis of the cell wall, nucleic acids, proteins, and folic acid.

Designing novel antibiotics against unexplored targets is one of the trending approaches in combating ABR. Recently, significant interest in the search for these novel targets has been mirrored by the increasing number of reports on the well-characterized bacterial cell division machinery.^2^ The divisome controls bacterial cell division, a dynamic multi-protein complex that synchronizes the partitioning of daughter chromosomes, localized cell wall production, and membrane invagination to produce a significant and efficient separation of daughter cells.^3^ The Filamentous Temperature Sensitive mutant Z (FtsZ) is regarded as the key cytokinesis-related protein within the divisome because it creates a “Z-ring” around the division site where the other proteins bind. Although the eukaryotic cytoskeletal protein tubulin and FtsZ share a high degree of structural similarity, there are significant structural differences between the two proteins, as well as differences in their GTP binding sites, polymerization characteristics, and protein partners.^4,5^ Their amino acid sequences also differ by less than 20%.^6^ This review presents not only the functions and crystal structures of FtsZ but also the known natural and synthetic inhibitors of FtsZ, emphasizing their mechanism of action and antibacterial activity. In addition, we also described the available *in vitro* detection and quantification methods for determining GTPase activity and polymerization inhibition of FtsZ-drug binding complexes. Furthermore, we also highlighted the recently reported small molecule FtsZ inhibitors and future directions of the ideal candidate.

### 1.1. FtsZ crystal structure

**Fig. 1:**
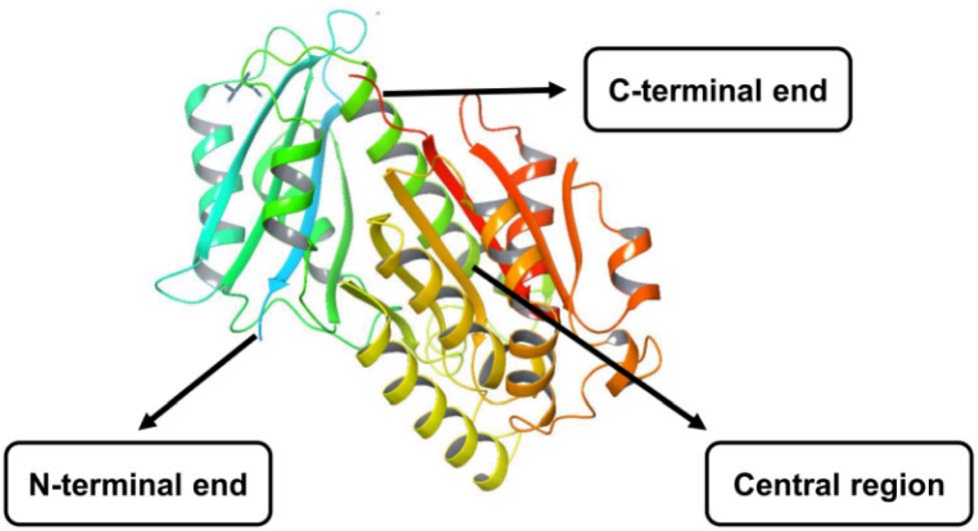
X-crystal structure of FtsZ protein (PDB ID: 6KVP).

The FtsZ protein, encoded by the *ftsz* gene, has a relative molecular weight of 40 kDa and has a GTPase. It binds to guanosine 5′-triphosphate (GTP) or guanosine 5′-diphosphate (GDP) in the presence of K^+^ and Mg^2+^ ions.^7^ Depending on its linkage with GTP or GDP, FtsZ occurs as monomers or higher-order polymers. It comprises two subglobular domains, N and C terminal, with diverse folds. The N-terminal domain connects to a C-terminal region with various extensions and is separated by the central core H5 and H7 helix. Both the domains were housed, each with a nucleotide-binding pocket and GTPase-activating site.^8,9^ In addition, the lengthy C-terminal tail interacts with various accessory proteins and is crucial for developing the FtsZ protofilament (Fig. 1).

In RCSB-PDB, almost 2176 crystal structures of FtsZ, with or without bound ligand were available from X-ray diffraction or NMR spectroscopy methods (Table 1). Availability of these crystal structures lead to the development of novel inhibitors against FtsZ protein.

**Table 1:** Dossier on X-ray crystal structures of FtsZ^a^

Pdb ID	Organism	Resolution (Å)	Ligand	References
6RVP	*S. aureus*	1.1	1-Methylpyrrolidin-2-one (MB3)	Heucas S. *et al.*, 2020 (ref. 42)
5XDT	*S. aureus*	1.3	1-Methylpyrrolidin-2-one (MB3)	Heffron T. P. *et al.*, 2017 (ref. 43)
6KVP	*S. aureus*	1.4	3-[(1*R*)-1-[5-Bromanyl-4-[4-(trifluoromethyl)phenyl]-1,3-oxazol-2-yl]-2,6-bis(fluoranyl)]benzamide (ZI1)	Ferrer-GonzálezE. *et al.*, 2019 (ref. 44)
2R75	*A. aeolicus*	1.4	8-Morpholin-4-ylguanosine-5′-(tetrahydrogen triphosphate) (01G)	Läppchen T. *et al.*, 2008 (ref. 45)
7OHH	*S. aureus*	1.4	Beryllium trifluoride ion (BEF)	Ruiz F. M. *et al.*, 2022 (ref. 46)
5MN4	*S. aureus*	1.5	(4*S*)-2-Methyl-2,4-pentanediol (MPD)	Wagstaff J. M. *et al.*, 2017 (ref. 47)
6YD5	*S. aureus*	1.5	3-[(3-Chlorophenyl)methoxy]-2,6-bis(fluoranyl)benzamide (OM8), 1-methylpyrrolidin-2-one (MB3)	Heucas S. *et al.*, 2021 (ref. 48)
6KVQ	*S. aureus*	1.6	[(2*R*)-2-[3-Aminocarbonyl-2,4-bis(fluronyl)phenoxy]-2-[5-bromanyl-4-[4-(trifluoromethyl)phenyl]-1,3-oxazol-2-yl]ethyl]3-[2,2-bis(fluoranyl)-10,12-dimethyl-3-aza-1-azonia-2-boranuidatricyclo[7.3.0.0̂^49^]dodeca-1(12),4,6,8,10-pentaen-4-yl]propanoate (DVX)	Gonzalez F. *et al.*, 2019 (ref. 44)
5XDV	*S. aureus,* subsp. aureus MRSA252	1.7	3-[[5-Bromanyl-4-[4-(trifluoromethyl)phenyl]-1,3-oxazol-2-yl]methoxy]-2,6-bis(fluoranyl)benzamide (Z16)	Fujita J. *et al.*, 2017 (ref. 50)
6YD1	*S. aureus*	1.7	2,6-Difluoro-3-methoxybenzamide (OLQ)	Heucas S. *et al.*, 2021 (ref. 48)
2RHJ	*B. subtilis*	1.7	Tetraethylene glycol (PG4)	Lovell S. *et al.*, 2009 (ref. 51)
1RQ2	*M. tuberculosis*	1.8	Citric acid (CIT)	Leung A. K. *et al.*, 2004 (ref. 52)
6SI9	*S. aureus*	1.9	1,2-Ethanediol (EDO)	Huecas S. *et al.*, 2020 (ref. 42)
4DXD	*S. aureus*	2.0	3-[(6-Chloro[1,3]thiazolo[5,4-*b*]pyridin-2-yl)methoxy]-2,6-difluorobenzamide (9 PC)	Tan C. M. *et al.*, 2012 (ref. 53)
6Y1V	*M. tuberculosis*	2.4	4-Hydroxy-2*H*-chromoen-2-one (4HC) dimethyl sulfoxide (DMS)	Alnami A. *et al.*, 2021 (ref. 54)
3WGN	*S. aureus*, subsp. aureus Mu50	2.6	5′-Guanosine-diphosphate-monothiophosphate (GSP)	Matsui T. *et al.*, 2014 (ref. 55)
3VOB	*S. aureus*, subsp. aureus Mu50	2.7	3-[(6-Chloro[1,3]thiazolo[5,4-*b*]pyridin-2-yl)methoxy]-2,6-difluorobenzamide	Yamane J. *et al.*, 2012 (ref. 56)

a Note: protein data bank (PDB), *Staphylococcus aureus* (*S. aureus*), *Aquifex aeolicus* (*A. aeolicus*), *Bacillus subtilis* (*B. Subtilis*), *Mycobacterium tuberculosis* (*M. tuberculosis*).

Further, screening techniques based on computational approaches and bioinformatics have been used to gain insights into reactions, bioavailability, and protein-ligand interactions.^10^ Designing, evaluating, comparing, modeling, predicting binding energies, pharmacokinetic and pharmacodynamic predictions, and the process of lead optimization have all gotten significantly simpler with the development of computational approaches.^11^ As an illustration of bioinformatics approaches, in 2023, Lu *et al.* demonstrated potentially effective synergistic combination of vancomycin and CEL (celastrol) for the treatment of VRE (vancomycin-resistant enterococcus) infections. Using the software Discovery Studio 4.5, molecular docking was carried out between CEL and FtsZ. With a perfect docking score of 113.1, CEL docked perfectly in the binding pocket of the FtsZ protein (PDB code: 5MN4). Overall, CEL may provide an innovative treatment alternative for treating VRE and can replace vancomycin as an antibacterial and adjuvant.^12^

### 1.2. Background and availability of FtsZ protein

In the 1960s, researchers looked for temperature-sensitive mutations that prevented cell division in *E. coli* at 42 °C. At 30 °C, the mutant cells usually multiplied but could not divide at 42 °C, and instead, long filamentous cells were formed by continuous growth without division (filamenting temperature sensitive). Several similar mutations were identified and assigned to a locus initially termed ftsA, which may include one or more genes. Lutkenhaus and Donachie demonstrated in 1980 that many of these mutations mapped to the same gene, ftsA,^13^ while one well-characterized mutant, PAT84, identified by Hirota *et al.*, mapped to a distinct, neighboring gene. This cell division gene was given the name *ftsZ*.^14^

Later on, with the advent of genomic technology, it became clear that FtsZ is mostly conserved throughout the bacteria and archaea species,^15^ with just a few outliers, such as the phylum Crenarchaeota, Planctomycetes, Chlamydiae,^16–18^ or the strains of *Carsonella ruddii*,^19^*Ureaplasma urealyticum*^20^, and *Mycoplasma mobile*.^21^ Furthermore, FtsZ also plays a major role during plastid division in algae and plants.

### 1.3. Functions of FtsZ

FtsZ is recognized as the key actor and pace-setting protein in the cell division of bacteria. The first and foremost protein to move to the mitotic locus is FtsZ.^22^ FtsZ self-polymerizes in a GTP-dependent way to form protofilaments and large bundles, ultimately assembling into a discontinuous ring-like structure at the inner side of the cytoplasmic membrane marking the potential site of division. Before the Z-ring starts to contract, additional proteins required for cell division are drawn into the cell center, where a septum is produced. By completing the Z-loop, one mother cell is split into two daughter cells, in which FtsZ stays in the cytoplasm as a monomer. New cell walls are then created between dividing cells. The Z-ring acts as a cytoskeleton, attracting at least 12 downstream cell division proteins to create the divisome complex, which may compress the cell membrane and form a septum between the cells.^23^ Actin-associated proteins including Serine protease F (SepF), zinc and iron regulated transporter like protein A (ZipA), and filamentous temperature sensitive mutant A (FtsA), which are found in Gram-positive bacteria, help to speed up the assembly of FtsZ.^24^ In the early/mid stage of cell division and septum development, Z-ring associated protein ZapA-D and SepF are crucial in regulating Z-ring dynamics. Negative regulatory proteins including mother cell inhibitor of Z (MciZ), sulfonamide resistant A (SulA), processive diacylglycerol β-glucosyltransferase (UgtP), extra Z-ring A (EzrA), caseinolytic protease X (ClpX), and minicell C (MinC) can stop FtsZ polymerization or postpone the production of Z-rings until the cell reaches a certain length.^25–28^

## 2. FtsZ inhibitors

The majority of anti-FtsZ drugs really disrupt FtsZ polymerization by obstructing critical GTP binding or FtsZ subunit interactions sites.^2,29^ Another approach could be to encourage or hyperstabilize the bundling of FtsZ polymers, which would disturb the dynamics of the polymer and lock it in an unusable form.^29^ The heterotypic associations of FtsZ with other proteins and its indirect adherence to the membrane are other approaches for FtsZ inhibition which are still under investigation. However, study by Silber *et al.*, 2020 exemplified that inhibition of these protein–protein interactions can combat resistance.^2,30^

The suppression of these interactions would also be more species-specific since FtsZ's binding partners are not well conserved. The other approach to inhibit FtsZ is to target its apparent propensity during crowding-induced dynamic condensation. It's interesting to note that condensate creation frequently occurs in cells expanding under stress, which coincides with the appearance of persister (a subpopulation of dormant cells that endure antibiotic treatment). Henceforth, inhibiting FtsZ might also acts as potential target for bacterial persistence.^31,32^

It has been established that a variety of FtsZ inhibitors, including, natural products, peptides and synthetic small compounds, can cause bacterial cell death by interacting with the several regions such as the nucleotide-binding pocket at the N-terminal region, the gap positioned between H7 and C terminus, and the C terminus of FtsZ.^23,33,34^

### 2.1. Natural compounds

Natural substances are a significant source of potential antibacterial agents; nearly 60% of medicines are derived from natural sources. Some FtsZ inhibitors are produced from natural compounds, such as berberine, curcumin, totarol and *etc*^35^ (Table 2).

**Table 2:** Dossier on natural compounds as FtsZ inhibitors^a^

Natural compounds	Biological source	Mechanism of action on FtsZ	Targeted bacteria	References
Curcumin	*Curcuma longa*	Increases the GTPase activity of FtsZ	Gram-negative and Gram-positive bacteria	Anand P *et al.*, 2008 (ref. 36), Kaur S. *et al.*, 2010 (ref. 38)
Cinnamaldehyde	*Cinnamomum vernum*	Inhibits FtsZ polymerization and GTPase activity	Both Gram-positive and Gram-negative bacteria	Domadia *et al.*, 2007 (ref. 39), Li *et al.*, 2015 (ref. 40)
Coumarins	Plants	Inhibits FtsZ polymerization and GTPase activity	*E. coli* and *M. tuberculosis*	Kontogiorgis C. *et al.*, 2012 (ref. 41), Duggirala *et al.*, 2014 (ref. 57), Zang *et al.*, 2020 (ref. 58)
Berberine	*Berberis* plants	Inhibits FtsZ polymerization and GTPase activity	*M. tuberculosis*, MRSA-MDR strains, Gram-positive and Gram-negative bacteria	Domadia P. N. *et al.*, 2008 (ref. 59), Stokes *et al.*, 2014 (ref. 49), Sun *et al.*, 2014 (ref. 60)
Totarol	*Podocarpus totara*	Inhibits GTPase activity and FtsZ assembly	Gram-positive bacteria, *M. tuberculosis*	Kim B. *et al.*, 2012 (ref. 61)
Plumbagin	*Plumbago zeylanica*	Inhibits Z-ring formation and decreases FtsZ protofilament formation along with suppression of FtsZ assembly	*B. subtilis*, *M. smegmatis*	Bhattacharya A. *et al.*, 2013 (ref. 62)
Sanguinarine	*Sanguinaria canadensis*	Inhibits protofilament bundling and assembly of FtsZ	*E. coli* and *B. subtilis*	Hemaiswarya S. *et al.*, 2011 (ref. 63), Wolff J. *et al.*, 1993 (ref. 64)
Viriditoxin	*Aspergillus viridinutans*	Inhibits FtsZ polymerization and GTPase activity	Broad-spectrum activity against drug resistance and sensitive Gram-positive bacteria	Suzuki K. *et al.*, 1990 (ref. 65), Wang J. *et al.*, 2003 (ref. 66)
Doxorubicin	*Streptomyces peucetius*	Suppresses Z-ring formation and GTPase activity	*E. coli*	Panda P. *et al.*, 2015 (ref. 67)
Dichamanetin	*Uvaria chamae*	Inhibits FtsZ polymerization and affects GTPase activity	Active against Gram-positive bacteria	Urgaonkar S. *et al.*, 2005 (ref. 68)

a Note: *Escherichia coli* (*E. coli*), *Mycobacterium tuberculosis* (*M. tuberculosis*), methicillin-resistant *staphylococcus aureus* multi-drug resistance (MRSA-MDR), *Mycobacterium smegmatis* (*M. smegmatis*), *Bacillus subtillis* (*B. subtilis*).

#### 2.1.1. Curcumin

**Fig. 2:**
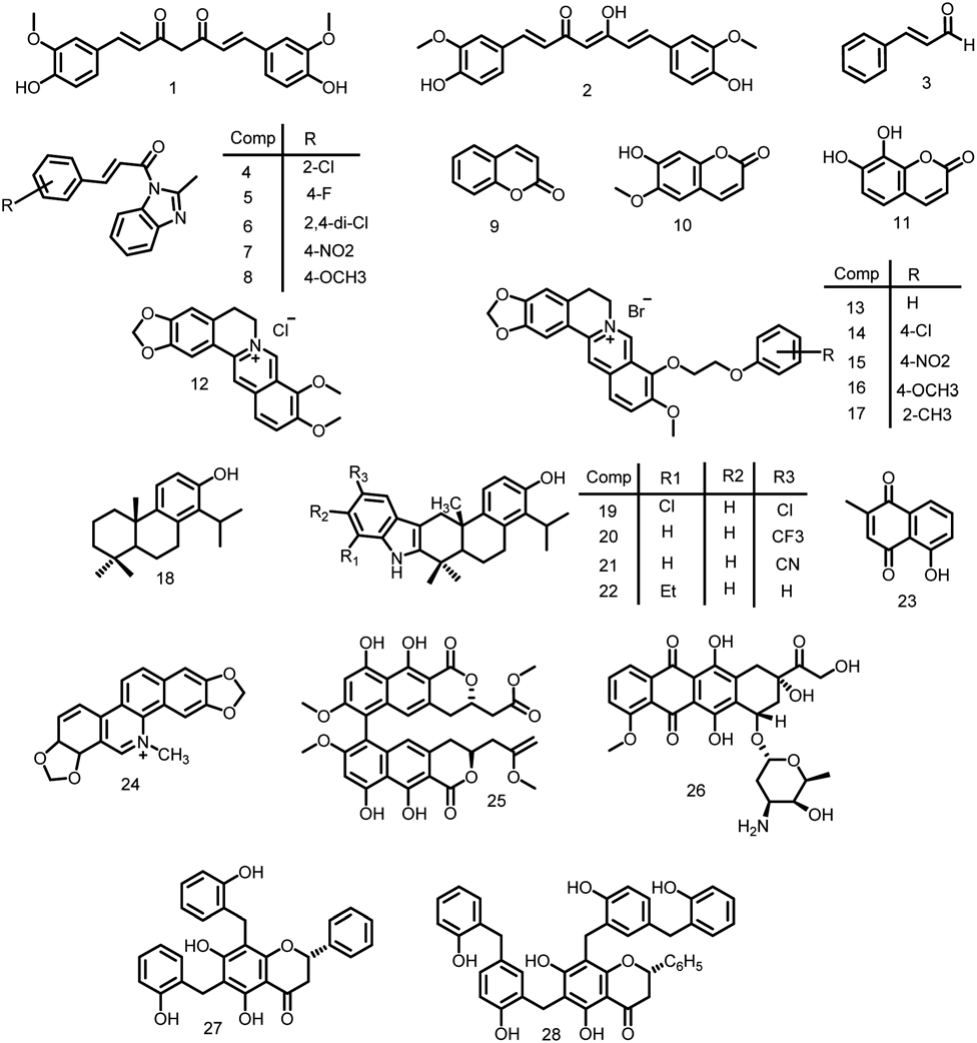
Chemical structures of natural compounds, 1-2: curcumin tautomeric forms; 3: cinnamaldehyde; 4–8: cinnamaldehyde derivatives, 9: coumarin; 10: scopoletin; 11: daphnetin; 12: berberine; 13–17: berberine derivatives; 18: totarol; 19–22: indolotoral derivatives; 23: plumbagin; 24: sanguinarine; 25: viriditoxin; 26: doxorubicin; 27: dichamanetin; 28: 2′′-hydroxy-5′′-benzyliso-uvarinol-B, active against FtsZ.

A naturally occurring dietary polyphenolic substance called curcumin that is derived from the rhizomes of the *Curcuma longa* plant has strong antibacterial properties against both Gram-positive and Gram-negative bacteria. The hydrophobic polyphenol curcumin [1, 7-bis (4-hydroxy-3- methoxyphenyl)-1, 6-heptadiene-3, 5-dione/diferuloylmethane] and its tautomeric form are depicted in Fig. 2 (1-2).^36^

Curcumin inhibited the growth of both Gram-positive (*B. subtilis* 168) and Gram-negative (*E. coli* K12MG1655 and *E. coli* BL21) bacteria, with a MIC value of 100 μM. Inducing filamentation in *B. subtilis* 168 cells shows that it prevents cytokinesis without appreciably changing the separation and structure of the nucleoids, which suggests that it prevents bacterial growth. The instability of FtsZ protofilaments is shown by the fact that there was an increase of 35% GTPase activity of FtsZ in the presence of 30 μM curcumin.^37^ Furthermore, *in silico* studies, also proved that curcumin exhibited greater binding affinity with the FtsZ receptor with a FlexX scores 17.55 kcal mol^−1^ (*E. coli*) and −18.84 kcal mol^−1^ (*Bacillus subtilis*) as evidenced by Kaur S. *et al.*, 2010. For the purpose of creating more powerful curcumin analogues with higher stability and bioavailability, the binding interactions of curcumin deserve investigation.^38^

#### 2.1.2. Cinnamaldehyde and its derivatives

The primary component of cinnamaldehyde, a 3-phenylpropanoid chalcone, is obtained from the stem bark of *Cinnamomum vernum*. Cinnamaldehyde (3, Fig. 2) exhibited significant MIC values of 1 μg ml^−1^ for *E. coli*, 0.5 μg ml^−1^ for *B. subtilis*, and 0.25 μg ml^−1^ for methicillin-resistant *Staphylococcus aureus* (MRSA) using the microbroth dilution technique.^39^ Cinnamaldehyde binds to the binding pocket at the C-terminal region containing the T7 loop of FtsZ resulted in disruption of cytokinetic Z-ring formation which was predicted by nuclear magnetic resonance and an *in silico* docking model. Li *et al.*, 2015 developed a unique technique to design and synthesize a new library of cinnamaldehyde derivatives (4–8, Fig. 2) and screened against a broad-range of Gram-positive and Gram-negative bacteria. The newly synthesized compounds had MIC values ranging from 0.25–4 μg ml^−1^ against *S. aureus* ATCC25923. Particularly, the most effective activity was seen in cinnamaldehyde derivatives with a 2-methyl benzimidazolyl substitution at the 1-position and phenyl, 2-chlorophenyl, 4-fluorophenyl, 4-chlorophenyl, 2, 4-dichlorophenyl, or 4-nitrophenyl at the 3-position.^40^

#### 2.1.3. Coumarins

The class of lactones known as coumarins isolated from plants and has benzopyrone skeletal structure.^41^ It has a variety of biological properties, including analgesic, anti-inflammatory, antiviral, antimalarial, antimicrobial, antiviral, anticoagulant, antioxidant *etc.* Duggirala *et al.*, 2014 successfully inhibited the GTPase and polymerization against the *E. coli* FtsZ protein by screening several coumarin analogues.^57^ Coumarins (9, Fig. 2), such as scopoletin (10, Fig. 2), inhibited FtsZ polymerization most effectively, with an IC_50_ of about 41 μM, followed by daphnetin (11, Fig. 2) (72 μM). Both compounds inhibited GTPase activity, with IC_50_ values of 23 μM and 57 μM, respectively.^58^

#### 2.1.4. Berberine and its derivatives

An alkaloid called berberine was discovered in numerous *Berberis* plants. It demonstrates antibacterial action against a wide range of bacteria, including several pathogenic species including *M. tuberculosis* and MRSA multidrug-resistant (MDR) strains. Domadia *et al.*, 2008 presented a series of biological experiments to examine the mechanism of berberine (12, Fig. 2). Using light-scattering & GTP hydrolysis assays, they discovered that berberine reduced both FtsZ polymerization (IC_50_ 10 ± 2.5 μM) and GTPase activity (IC_50_ 16.01 ± 5 μM).^59^ An *in silico* docking analysis using AutoDock software found that the interaction of berberine's dimethoxy groups, isoquinoline nucleus, and benzodioxole ring with FtsZ, which provided the probable binding site, was consistent with the saturation transfer difference-non-magnetic resonance (STD NMR). Sun *et al.*, 2014 created a series of 9-phenoxy alkyl berberine derivatives (13–17, Fig. 2) as effective FtsZ inhibitors by combining *in vitro* bioassays with *in silico* structure-based design.^60^

#### 2.1.5. Totarol

Totarol, a diterpenoid phenol (18, Fig. 2) isolated from *Podocarpus totara*, a plant endemic to New Zealand, induces filamentation in *B. subtilis* cells and inhibits bacterial cytokinesis. Totarol had no noticeable effects on the nucleoid segregation or the membrane structure in *Bacillus subtilis* cells at its lowest effective concentration (1.5 μM). However, destruction of the cytokinetic Z-ring indicates that at the same dose, it hinders bacterial cytokinesis by disrupting the development and function of the Z-ring. Totarol was discovered to bind to *Mtb*FtsZ with a low affinity (*K*_d_), 11 ± 2.3 μM and hinders protofilament assembly and GTPase activity through altering the protein's structure. It may serve as a lead molecule for the creation of a FtsZ targeting inhibitors because it suppressed the GTPase activity of isolated *Mtb*FtsZ by 50% (40 μM totarol) and hindered the growth of *B. subtilis* at a MIC of 2 μM. Kim *et al.*, 2012 described the synthesis of totarol's heterocyclic analogues that led to the development of indolototarol derivatives (19–22, Fig. 2) with improved antibacterial activity.^61^

#### 2.1.6. Plumbagin

Plumbagin, also known as 5-hydroxy-2-methyl-1,4-naphthoquinone, is a secondary plant metabolite that is obtained from the roots of *Plumbago zeylanica*. It has a number of biological properties, including the ability to prevent the growth of mammalian, fungus, and bacterial cells. According to Bhattacharya *et al.*, 2013 plumbagin (23, Fig. 2) prevented the proliferation of *Mycobacterium smegmatis* and *B. subtilis* 168 cells. It also inhibited the development of a functioning Z-ring and increased the cell length of *B. subtilis* 168, eventually suppressed its cell growth in a concentration-dependent way. In addition, it was observed that in the presence of 2, 5, and 10 μM plumbagin, the extent of the protofilament assembly was decreased by 26, 33, and 45%, respectively. The plumbagin binding site was close to the C-terminal of *B. subtilis* FtsZ (BsFtsZ), according to docking research, and it interacted with BsFtsZ *via* hydrophobic and hydrogen-bonds. Furthermore, with the help of docking analysis it was discovered that two BsFtsZ residues, Asp199 and Val307 were crucial for the binding interactions between plumbagin and BsFtsZ. The findings provide support for the development of powerful plumbagin analogues because of its bactericidal property *via* preventing FtsZ assembly.^62^

#### 2.1.7. Sanguinarine

A polycyclic alkaloid called sanguinarine (24, Fig. 2), which is produced from the rhizomes of *Sanguinaria canadensis*, inhibits the formation of FtsZ protofilaments by reducing FtsZ polymerization.^63^ Beuria and colleagues in the year 1993 showed that sanguinarine prevented cytokinesis in both Gram-positive and Gram-negative bacteria by inhibiting the assembly of the *E. coli* FtsZ. With context to *B. subtilis* 168, *E. coli* BL21 (wild-type), and *E. coli* JM109 (WM647), the IC_50_ values of sanguinarine were 1.0 ± 0.3, 4.6 ± 0.8, and 12.0 ± 1.7 μg ml^−1^, respectively. Additionally, it has the ability to block eukaryotic tubulin, suggesting that it affects mammalian cells. Because of its non-selective nature, the development of sanguinarine derivatives were of least concern.^64^

#### 2.1.8. Viriditoxin

*Aspergillus viridinutans* derived viriditoxin was initially discovered in 1971, but its structure was mistakenly ascribed at the time and was later rectified in 1990 (25, Fig. 2).^65^ Viriditoxin was isolated as a FtsZ inhibitor from a pool of over 100 000 extracts of microbial fermentation broths and plants, which were then fractionated using a fluorescent FtsZ polymerization assay. It was demonstrated to inhibit concurrent GTPase inhibition with an IC_50_ of 7.0 μg ml^−1^ and block *E. coli* FtsZ polymerization with an IC_50_ of 8.2 μg ml^−1^. However, viriditoxin lacks the capacity to inhibit the synthesis of DNA, RNA, proteins, fatty acids, or cell walls as evident by Wang *et al.*, 2003. In addition, viriditoxin displayed broad-spectrum antibacterial action against a wide range of clinically relevant pathogens, indicating that FtsZ is highly functionally conserved in these species.^66^

#### 2.1.9. Doxorubicin

Doxorubicin (26, Fig. 2) was selected from a pharmacological library that has been authorized by the U.S. FDA utilizing an independent computational, molecular, and microbiological strategy to find small compounds that target FtsZ and prevent bacterial division. Doxorubicin, an anthracycline antibiotic derived from the actinobacterium *Streptomyces peucetius*, has been found to be a powerful FtsZ inhibitor and can suppress the development of *E. coli* by disrupting FtsZ functions. A study by Panda *et al.*, 2015 revealed the antibacterial susceptibility of doxorubicin for the various bacterial strains. The fluorescence-binding experiment demonstrates that doxorubicin significantly interacts with FtsZ in bacteria without altering membrane composition or nucleoid segregation. Doxorubicin obviously suppresses Z-ring formation and consequently cell division in *E. coli*, as evidenced by the finding that the number of correct Z-rings per cell was 0.95 ± 0.1, 0.8 ± 0.2, and 0.2 ± 0.8 in the absence or presence of 20 μM and 40 μM doxorubicin, respectively. Doxorubicin at a 10 μM concentration decreased light scattering intensity by around 25% while inhibiting GTPase activity by 27%, indicating that the two outcomes are equivalent. Also, doxorubicin has similar effects on FtsZ assembly and GTPase activity as evident by their *K*_m_ values for sedimentation (0.49 M) and GTPase activity (0.72 M). Furthermore, a number of single amino acid changes at the discovered binding site in FtsZ resulted in a several-fold drop in FtsZ doxorubicin affinity, demonstrating the relevance of this region for doxorubicin interaction. The discovery of a novel binding site can be enhanced in order to identify newer and more effective FtsZ-targeted antibacterial agents.^67^

#### 2.1.10. Dichamanetin

The two naturally occurring polyphenolic substances-dichamanetin (27, Fig. 2) and 2′′′-hydroxy-5′′-benzylisouvarinol-B, separately identified by Hufford *et al.*, 1979 and Anam from *Uvaria chamae* and *Xylopia afticana*, respectively, are strong inhibitors of the GTPase activity of FtsZ. Using a novel zinc chloride-mediated benzylic coupling process, these two natural compounds, dichamanetin and 2′′′-hydroxy-5′′-benzylisouvarinol-B, were produced from a similar core structure. *E. coli* GTPase activity is effectively inhibited by both substances. The IC_50_ values of dichamanetin (12.5 ± 0.5 M) and 2′′′-hydroxy-5′′-benzyliso-uvarinol-B (8.3 ± 0.5 M) indicated that they are active against the bacterial cell division protein FtsZ.^68^

#### 2.1.11. Antimicrobial peptides or AMPs

Antimicrobial peptides or AMPs are highly promising molecules that exhibit excellent antibacterial activity and can be considered as a potential alternative to conventional antibiotics. Several experiments have demonstrated that peptides have successfully killed 86 multidrug resistant (MDR), extensively resistant (XDR), and pandrug resistant (PDR) bacteria.^87^ These small peptides either occur naturally as a part of host defense mechanism or can be synthesized. Peptides can be classified on the basis of source, activity, structural characteristics and amino-acid rich species.^88^ Stronger analogues of natural products produced using the structure and activities of peptides are called peptidomimetics as they are the consequent molecular imitators.^89^ Some of the peptides (Table 3) exhibiting potent activity against FtsZ, are mentioned below:

**Table 3:** Dossier on peptides as FtsZ inhibitors^a^

Peptide inhibitors	Mode of action	Targeted bacteria	References
MciZ	Inhibition of FtsZ assembly	*B. Subtilis*	Araújo-Bazán *et al.*, 2016 (ref. 69), Ray S. *et al.*, 2013 (ref. 70)
CRAMP	Inhibition of FtsZ assembly and GTPase activity	*B. Subtilis* and *E. coli*	Ray S. *et al.*, 2014 (ref. 71)
Edeine	Inhibition of FtsZ assembly and bacterial DNA/protein synthesis	*B. Subtilis*	Ray S. *et al.*, 2014 (ref. 71), Shimotohno K. W. *et al*., 2010 (ref. 72)
Kil	Inhibition of GTPase activity and the Z-ring formation	*E. coli*	Bi E. *et al.*, 1993 (ref. 73), Haeusser D. P. *et al.*, 2014 (ref. 74)
FtsZps	Inhibition of FtsZ assembly and GTPase activity	*E. coli*	Paradis-Bleau *et al.*, 2004 (ref. 75)
NCR247	Inhibition of FtsZ polymerization and formation of Z-ring and septum	*S. meliloti*	Van de velde *et al.*, 2010 (ref. 76), Farkas *et al.*, 2014 (ref. 77)
ADEPs	FtsZ degradation and prevention of Z-ring formation	MRSA and *S. pneumoniae*	Sass P. *et al.*, 2011 (ref. 78), Clement J. *et al.*, 2005 (ref. 79)
I19L	Inhibition of FtsZ bundling assembly	*E. coli*	Clement J. *et al.*, 2009 (ref. 80)
N2/N6	Suppression of Z-ring formation and FtsZ assembly	*E. coli* and *S. enteritidis*	Farkas *et al.*, 2014 (ref. 77 and 81)
PNAs	Inhibition of the *ftsZ* gene expression	*S. aureus*, *E. coli* and MRSA	Ghosal *et al.*, 2013, Good L. *et al.*, 2001, Liang S. *et al.*, 2015 (ref. 82–84)
TL	Suppression of GTPase activity of FtsZ	*E. coli*, Gram positive and Gram negative bacteria	Somma A. D. *et al.*, 2020 (ref. 85), Somma A. D. *et al.*, 2021 (ref. 86)

a Note: mother cell inhibitor of Z (MciZ), *Bacillus subtilis* (*B. subtilis*), cathelin-related antimicrobial peptide (CRAMP), *Escherichia coli* (*E. coli*), node-specific cysteine-rich (NCR), *Sinorhizobium meliloti* (*S. meliloti*), acyldepsipeptides (ADEPs), methicillin-resistant *staphylococcus aureus* (MRSA), *Streptococcus pneumoniae* (*S. pneumonia*), *Salmonella enteriditis* (*S. enteritidis*), peptic nucleic acid (PNAs), *Staphylococcus aureus* (*S. aureus*), temporin L (TL).

##### 2.1.11.1. MciZ: negative regulatory protein

The negative regulatory peptide MciZ, which comprises of 40 amino acid residues, is an intrinsic inhibitor that activates during spore formation to suppress Z-ring formation in mother cells by disrupting FtsZ assembly.^69^ MciZ mostly attach at C-terminal polymerization interface of *B. subtilis* FtsZ. By blocking FtsZ polymerization through steric hindrance, MciZ shortens FtsZ protofilaments and enhances the particular GTPase activity.^70^*B. subtilis* MciZ expression was seen during spore production, according to research by Handler *et al.*, 2008 on the effects of MciZ on Z-ring development.^26^ In a molecular dynamics (MD) simulation study by Bisson-Filho *et al.*, 2015 demonstrated that the MciZ–FtsZ interaction require a salt-bridge between the guanidinium group of Arg20 of MciZ and the carboxylate of Asp280 of *B. subtilis* FtsZ. The Asp280-Arg20 salt bridge is extremely important for complex stability, and mutations in FtsZ (from Asp → Arg at 280th position) or MciZ (Arg → Asp at 20th postion) could disrupt the MicZ–FtsZ interaction. Additionally, hydrogen bonds formed between β9 of FtsZ and β2 of MciZ, as well as hydrophobic interactions between helices H1 of MciZ and H10 of FtsZ, could stabilize the interaction.^90^ Further various approaches including hydrogen–deuterium exchange and fluorescence correlation spectroscopy were used to demonstrate that MciZ can sequester FtsZ monomers, affect FtsZ's conformation, and block the polymerization interface at the (+)-end of FtsZ filaments, thereby hindering treadmilling dynamics and inducing filament disassembly.^91^

##### 2.1.11.2. CRAMP: a murine AMP

In various mammals, cathelicidins are the precursors for potent AMPs. A cathelin-related antimicrobial peptide (CRAMP), with 37 amino acid residues was initially identified in the murine bone marrow and neutrophils. CRAMP plays a key function in host defensive response and can modulate innate immunity. The CRAMP (16–33) peptide (GEKLKKIGQKIKNFFQKL) targets FtsZ to inhibit Z-ring formation, stimulate cell length elongation, and inhibit the growth of *B. subtilis* and *E. coli* (with MICs of 20 μM) as proven by Ray *et al.*, 2014. The secondary structure of *B. subtilis* FtsZ was altered by creating a salt-bridge between Lys25 of CRAMP and Asp287 of FtsZ. *In vitro* FtsZ polymerization and GTPase activity were reduced by the peptide CRAMP (16–33) after it bonded to the C terminus of FtsZ close to the T7 loop.^71^

##### 2.1.11.3. Kil: a bacteriophage peptide

Bacteriophages are capable of infecting bacteria and finally hinder the cell division throughout the infection process. Although these viruses represent a potential weapon against pathogenic bacteria, it is still unclear how exactly their inhibitory properties affect the formation of the division ring at the molecular level. Kil peptide expressed from *kil* gene of *E. coli* bacteriophage λ, contains 47 amino acid residues that can block cell division in *E. coli* and causes bacterial cell filamentation.^74^ Kil interacted with FtsZ-GDP, inhibiting overall GTPase activity and effectively blocking Z-ring formation. Kil, like SulA, an inhibitor of FtsZ assembly, may cause the SOS response in *E. coli*, which inhibits cell division.^73,92^ In 2022, Dhanoa *et al.* employed fluorescent *E. coli* EV36/FtsZ-mCherry and K12/FtsZ-mNeon strains to evaluate the influence of bacteriophages on FtsZ using an *in vitro* meningitis model system. They demonstrated that FtsZ is normally localised to the bacterial cell midbody as a single ring. However, when the known inhibitor kil peptide is administered, FtsZ is mislocalized, resulting in filamentous multi-ringed bacterial cells.^93^

##### 2.1.11.4. Edeines

Edeines (subtypes A and B) are polypeptide antibiotics isolated from *Brevibacillus brevis* that may suppress *B. subtilis* cell growth (MIC of 20 μM) and Z-ring formation. These peptides were initially found in 1959 and have been shown to be effective against a number of species, including fungus, cancer cell lines, Gram-positive and Gram-negative bacteria. Edeine is classified into two subtypes: bioactive (edeine A1 and B1) and inactive (edeine A2 and B2) isomers.^72^ The active isomer edeine A1 hindered bacterial DNA and protein synthesis, whereas edeine B1 inhibited protein synthesis and cell division *via* inhibiting FtsZ assembly, enabling *B. subtilis* to take on a filamentous shape.^94^

##### 2.1.11.5. Acyldepsipeptides

Acyldepsipeptides (ADPEs) are a class of particular acyl peptides that have strong antibacterial activity with the MICs of 0.01–0.05 μg ml^−1^ against MDR staphylococci, streptococci, and enterococci both *in vitro* and *in vivo* (mice and dogs).^78^ In Gram-positive bacteria, ADEP inhibits cell division. It also causes severe filamentation in the rod-shaped *B. subtilis* and swelling in the coccoid *S. aureus* and *S. pneumoniae*. It was discovered that ADEP treatment suppresses septum development during the Z-ring assembly stage and that central cell division proteins move away from their mid-cell locations. Contrary to typical antibiotics that predominantly target protein, folic acid, DNA/RNA, or cell wall production, ADEPs have the ability to modulate the ATP-dependent casein-hydrolyzed protease ClpP to an uncontrolled state. The complex ADEP-ClpP, thus, inhibits *B. subtilis* FtsZ and prevents the Z-ring formation and eventually cell division.^79^

##### 2.1.11.6. FtsZps

Using phage display technique, the short peptide ligands, such as FtsZp1 (CSYEKRPMC), FtsZp2 (CLTKSYTSC), and FtsZp3 (GAVTYSRISGQY), were found in the random peptide libraries PH.D.-12 (2.7 × 10^9^ 12-mer sequences), and PH.D.-C-7-C (3.7 × 10^9^ 12-mer sequences). The inhibitory capacity results revealed that these three synthesized peptides could specifically inhibit FtsZ GTPase activity. Further, the study by Paradis-Bleau C. *et al.*, 2004 clearly exemplified that both the FtsZp1 and FtsZp2 (the C-7-C-mer peptides) possesses higher affinity for *P. aeruginosa* FtsZ than FtsZp3 (12-mers), which may be because of the presence of disulfide bond in C-7-C-mer peptides.^75^

##### 2.1.11.7. N2 and N6: marine peptides

The marine environment contains roughly 10^6^ bacteria perml and 10^9^ viruses per ml of saltwater, making it a rich source of infections. Since marine organisms dwell in close proximity to microbial pathogens, they require a powerful and effective immune system to survive in such a hostile environment, and AMPs serve as the first line of defence against invading microbes. Marine AMPs have been demonstrated to be structurally distinct from their terrestrial analogues, and they frequently exhibit novel structures.^95^ The marine peptides N2 and N6, which have MICs of 0.125 to 1 g ml^−1^, exhibit potent antibacterial properties against *E. coli* and *S. enteritidis*. They are produced from NZ17074, an arenicin analogue isolated from the marine invertebrate lugworm *Arenicola marina*. N2 and N6 both damaged *E. coli*'s outer and inner membranes, triggered cell cycle arrest in phase I, and hindered the production of *E. coli* DNA, RNA, and cell wall. The outer membrane of *S. enteritidis* was penetrated by N2 and N6, the cell cycle stopped at phase R, and peptides hindered the production of DNA, RNA, and protein. Moreover, Yang N *et al.*, 2017 proposed that both N2 and N6 hindered cell division causing filamentation of *E. coli* which may be connected to the suppression of Z ring formation and FtsZ assembly, however, further investigation is required to validate the statement.^96^

##### 2.1.11.8. I19L: a stathmin family protein

Major cyto-skeletal segments called microtubules are essential in several cellular processes like mitosis, cell motility, and intracellular traffic. These dynamically assembled αβ-tubulin cylindrical polymers assemble in a highly controlled manner. The stathmin family proteins trap tubulin in a nonpolymerizable ternary complex *via* their stathmin-like domains (SLD) and thereby contribute to the regulation of microtubule dynamics. The short peptides produced from the N-terminus of SLDs inhibit tubulin polymerization with varying efficiencies, and that phosphorylation of the most potent of these peptides diminishes their efficacy, just like full-length stathmin.^79^ The short peptide I19L, which is generated from the N-terminal of the cytosolic phosphoprotein stathmin, can prevent the formation of microtubules by interacting with tubulin through the L17, L19, and F15 residues.^79^ Clement *et al.*, 2009, analyzed the I19L's impact on FtsZ polymerization and discovered that the hydrophobic residues (R78, E168, and R169) bonded to the GTP pocket or T7 loop of *E. coli* FtsZ and prevented FtsZ bundling assembly with the help of Ca^2+^ ions.^80^

##### 2.1.11.9. NCR247: a node-specific cysteine rich AMP

In various symbiotic systems, peptides obtained from the host that are specific to the symbiosis are in charge of controlling intracellular endo-symbiotic bacteria. Most of the activities of these peptides are unknown. Many legumes that have a facultative rhizobium-legume symbiosis have bacteria that develop into enormous polyploid, uncultivable bacteroids. Node-specific cysteine-rich antimicrobial peptides (NCR-AMP) are essential for bacteroids formation.^97^ In rhizobia-infected plants, over 600 NCR peptides have been isolated.^77^ NCRs, like defensin, are a prominent family of AMPs comprised of eight cysteines and a distinct signal peptide and have antibacterial action against various pathogens. One such peptide is the cationic peptide NCR247 (containing 24 amino acids) which had a low MIC of 5 M and demonstrated *in vitro* antibacterial activity against *Sinorhizobium meliloti*.^76,81,97^ It also penetrated bacterial cell membranes and formed interactions with several bacterial proteins. Farkas *et al.*, 2014 reported that NCR24 inhibited FtsZ polymerization by binding to FtsZ monomers, ultimately preventing *S. meliloti* from forming a Z-ring and septum. NCR247 interfered with bacterial cell division and led to cell elongation before FtsZ localised to the centre of the *S. meliloti* cell.^77^

##### 2.1.11.10. Peptide nucleic acids

Peptide nucleic acids (PNA) are analogues of oligonucleotides that, unlike nucleotides, have a skeleton that is more similar to a polypeptide than a ribophosphate. PNA interferes with ribosomal binding by targeting important genes, such as the ftsZ gene, which inhibits gene expression and causes bacterial cell death.^98^ PNA has substantial action against MRSA, *S. aureus*, and *E. coli* with MICs of 0.2–5 μM and may enter bacterial cells with the support of a carrier peptide.^82^ Few carrier peptides that can facilitate PNA entrance into bacterial cells are mentioned below:

(i) ***(RXR)4XB***: PNA can be delivered to cells through cell-penetrating peptides (CPPs), which include positively charged residues.^83^ Peptide (RXR)4XB, a CPP, is the most frequently used peptide in bacteria to enhance PNA entrance, suppress gene expression, and promote cell growth.^84,99^ Liang *et al.*, 2015 discovered that (RXR)4XB-conjugated PNAs targeting FtsZ, such as PPNA1 (targeting 309–323 nucleotides of the *ftsZ* gene) and PPNA2 (targeting the translation, initiation of *ftsZ* gene), could inhibit MRSA growth *in vitro via* dose-dependent down regulation of *ftsZ* gene expression.^84^ Narenji *et al.*, 2020 coupled (RXR)4XB PNA to the *Enterococcus faecalis* ftsZ and *efa*A genes, which are involved in biofilm formation and cell division, respectively. The results demonstrated that this combination suppressed *E. faecalis* cell division, proliferation, and biofilm formation. Furthermore, the peptide PNA exhibited no cytotoxicity against human MCF7 cells indicating greater selectivity towards the bacteria.^100^

(ii) ***(KFF)3K***: In both Gram-negative and Gram-positive bacteria, including *E. coli* and *S. aureus*, the (KFF)3K peptide demonstrates excellent potential as antisense reagent carriers.^82,99,101,102^ In order to target the ftsZ and acyl carrier protein *acp*P genes, Ghosal *et al.*, 2012 created L- or D-type (KFF)3K-PNA conjugates and screened for their antibacterial activity. The cell division (ftsZ) and fatty acid synthesis (*acp*P) genes in *Pseudomonas aeruginosa* were found to inhibited by the (KFF)3K-PNA conjugates.^103^

(iii) ***Others***: Other peptides, such as H-KKHRKHRKHRKH, H-D(KFFKFFKFFK), H-FWRIRIRR, H-(RFR)_4_-Ahx-βala, H-(R-Ahx-R)_4_-Ahx-βala, H-(R-Ahx)_6_-βala, and H-D((KFF)_3_K), were associated with the anti-FtsZ/*Acp*P PNAs. According to the findings, PNA only displayed strong antibacterial action against *P. aeruginosa* when conjugated with H-(R-Ahx)_6_-βala or the H-(R-Ahx-R)_4_-Ahx-βala.^103^ It also lowered the expression of the targeted ftsZ and *acp*P genes. The *E. coli* ΔsbmA strain could not proliferate, when the anti-FtsZ/*Acp*P-PNA was conjugated with these peptides.^82^

##### 2.1.11.11. Temporin L: an amphibian peptide

One of the largest families of AMPs with natural origin are the amphibian Temporins, which have potent antibacterial activities against a variety of Gram-positive and Gram-negative bacteria that causes infections in humans including skin disorders, meningitis, and urinary tract infections. Temporins were originally discovered in the skin of the Asian frog *Rana erythraea* and were initially referred to as Vespa-like due to their sequence resemblance to chemotactic and histamine-releasing peptides extracted from the venom of Vespa wasps. Later in 1996, Simmaco *et al.* discovered a family of 10 structurally similar peptides with antibacterial and antifungal activities in the skin secretions of the European common red frog *R. temporaria* when it was electrically stimulated, and named these peptides temporins, from A to L.^104^ Fmoc (flourenylmethoxy carbonyl) amino acids were used to synthesize the peptide analogues (biotin conjugated temporin L or TL and fluorescein conjugated TL) and purification was done using semi-preparative RP-HPLC (reverse phase high performance liquid chromatography). By observing *E. coli* cell growth at various TL concentrations, the antibacterial activity of TL was confirmed with the MIC value of 32 μM. The functional effects of TL on FtsZ were then investigated *in vitro* and *in vivo*. Enzymatic studies of FtsZ GTPase activity in the presence of TL (IC_50_ value −62 ± 2 μM) demonstrated that TL is a competitive inhibitor of the protein, as predicted by the docking simulation. The peptide crosses the bacterial outer membrane and specifically binds FtsZ, suppressing its GTPase activity by a competitive inhibition mechanism. Temporin L cannot be considered an effective alternative to conventional antibiotics due to its haemolytic activity, however optimization of the peptide characteristics by subtle chemical structure alteration can minimise its haemolytic activity.^85,86^

#### 2.1.12. Synthetic products

##### 2.1.12.1. Zantrins

**Fig. 3:**
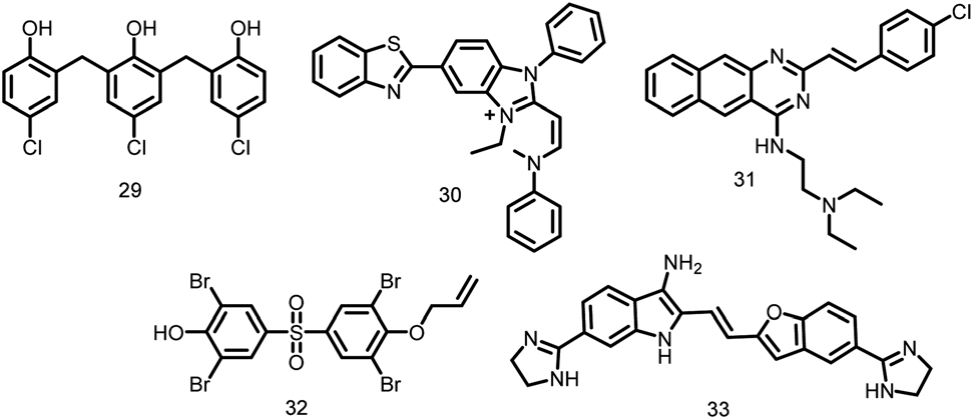
Chemical structures of zantrins (29–33).

Five different phenolic compounds known as zantrins (Fig. 3) collectively alter the GTPase activity of FtsZ (IC_50_ 4–100 μM) and either destabilize (29–30, 32) or promote hyperstability of FtsZ protofilaments by facilitating lateral linkages (zantrins 31 and 33). Except 33, all zantrins decrease the frequency of Z-ring formation without disturbing the do not cause filamentation of *E. coli*. In order to produce more promising lead compounds, zantrins need to be further optimized due to their relatively low antibacterial activity (MICs 66–98 μM).^105^

##### 2.1.12.2. Benzimidazoles

**Fig. 4:**
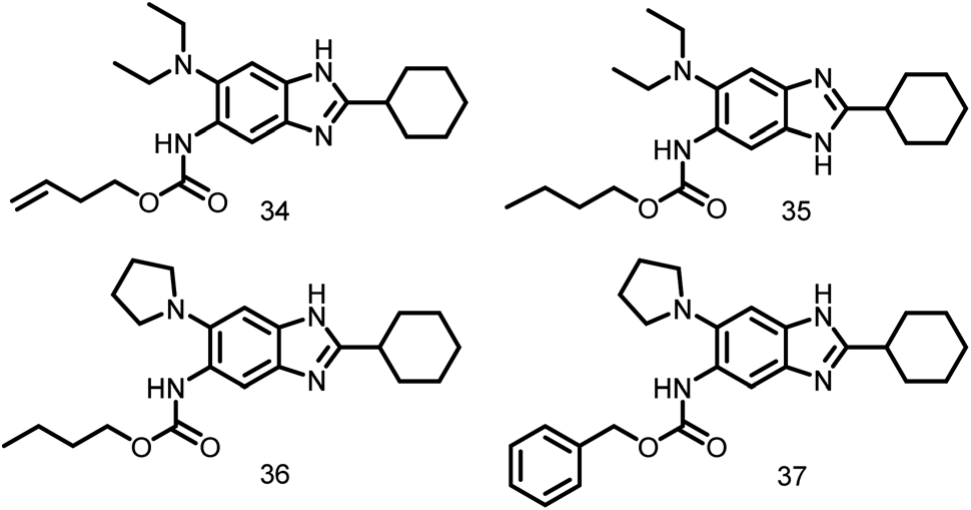
Chemical structures of benzimidazole derivatives.

From a 272-compound library, various novel trisubstituted benzimidazoles (34–37) were rationally designed (Fig. 4). These compounds exhibited excellent antibacterial activity against clinical *M. tuberculosis* (MICs of 2–15 μM) and *Francisella tularensis* (MIC_90_ of 0.35–48.6 g ml^−1^) as evident by Kumar *et al.*, 2011. Further, these tri-substituted benzimidazoles resulted in undivided cells by disrupting FtsZ assembly and Z-ring formation in dose-dependent fashion.^106^

##### 2.1.12.3. Benzamides

**Fig. 5:**
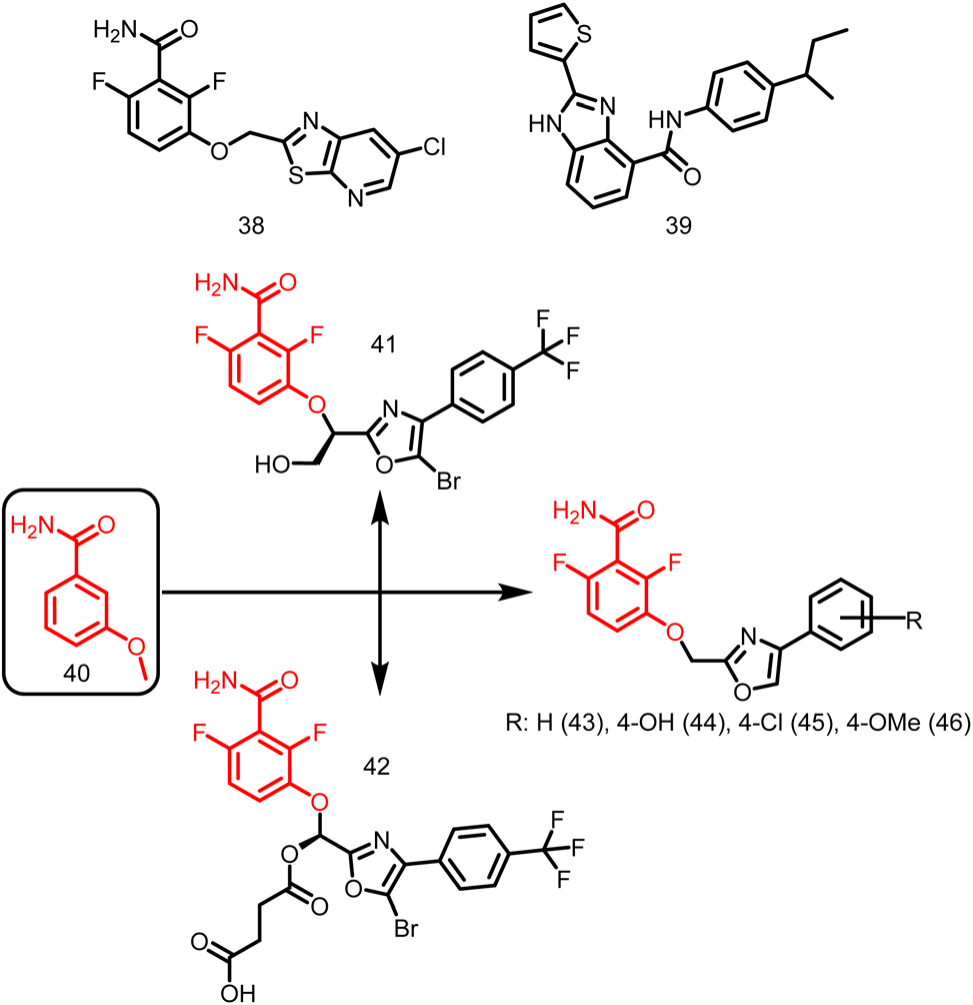
Chemical structures of benzamide derivatives.

PC190723 (calbiochem-benzamide ether derivative), (38, Fig. 5), one of a number of synthetic FtsZ-targeting antibacterial agents, has powerful bactericidal activity against a number of Gram-positive bacteria, including *B. subtilis*, MRSA, and other MDR *S. aureus*, both *in vitro* and *in vivo* by inducing filament assembly and preventing cell division. As per the research conducted by Ray *et al.*, 2015 the benzimidazole derivative, BT-Benzo-29 (39, Fig. 5) demonstrated strong antibacterial action against *B. subtilis via* hindering FtsZ assembly.^107^ Recently, Stokes N. *et al.*, 2013 reported another substituted 3-MBA (3-methoxybenzamides) derivative (40, Fig. 5) and its succinate pro-drug. The two compounds (41-42, Fig. 5) exhibited significant *in vitro* and *in vivo* activity as compared to the parent drug. The synthesis and characterization of phenyl oxazole moeity (43–46, Fig. 5) marked the beginning of their initial explorations into the oxazole series. Additionally, three additional 2, 4-substituted oxazoles were synthesized. Compound possessing –H (43) on the oxazole nucleus was shown to start suppressing cell division at around the same concentration and to inhibit wild-type *S. aureus* at a MIC of 16 μg ml^−1^ that is comparable with its capability to inhibit FtsZ activity in order to exert its antibacterial effect. Replacement of –H with –OH, –Cl, OCH_3_ (44–46) on the phenyl ring increased the antibacterial effectiveness.^49^

##### 2.1.12.4. Arene-diol digallates

**Fig. 6:**
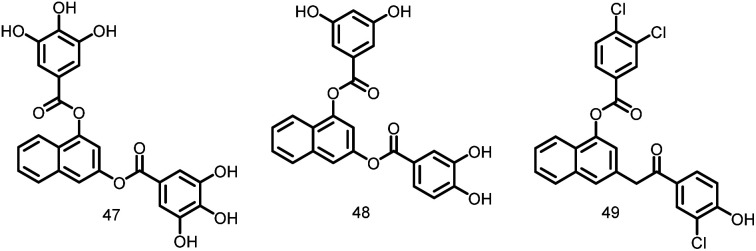
Chemical structures of arene-diol digallate derivatives (47–49).

Recently, Ruiz Avila *et al.*, 2013 reported a large number of novel active substances (47–49, Fig. 6) and screened by docking into the *B. subtilis* FtsZ GTP-binding site. These FtsZ inhibitors caused prolonged, undivided cells in *B. subtilis*, MDR *S. aureus*, and *E. faecalis* by causing a cascade of reactions including, substituting GTP, impairing Z-ring formation, delocalizing FtsZ into several foci, and inhibiting cell division. The majority of substances exhibit strong action against Gram-positive bacteria, such as MRSA (MIC: 7 μM), and low cytotoxicity against mammalian cells, suggesting the possibility of attractive therapeutic candidates in the future.^108^

##### 2.1.12.5. Taxane

**Fig. 7:**
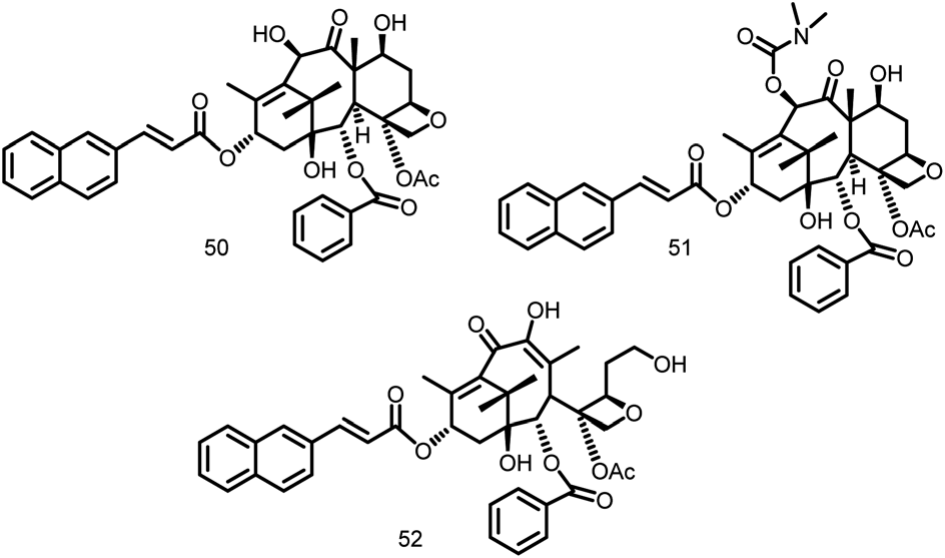
Chemical structures of taxane derivatives (50–52).

The drug taxane was examined to identify both highly cytotoxic taxoids that stabilize microtubules and noncytotoxic (or very mildly cytotoxic) taxane-multidrug-resistance (MDR) reversal agents (TRAs) that block the efflux pumps of ATP-binding cassette (ABC) transporters like P-glycoprotein. Huang *et al.*, 2006 reported the identification of 120 taxanes with remarkable antituberculosis activity. The rational optimization of the chosen substances allowed for the identification of the noncytotoxic nature of C-seco-taxane multidrug resistance (MDR) reversal agents (C seco-TRAs). In a light-scattering assay, it was discovered that C-seco-TRA stabilizes FtsZ protofilaments of *M. tuberculosis* cells, acting similarly to paclitaxel as an anticancer drug that encourages tubulin formation and maintains microtubules. MIC99 values for these noncytotoxic taxane lead compounds (50–52, Fig. 7) ranged from 1.25 to 2.5 μM for both drug-resistant and drug-sensitive *M. tuberculosis* strains.^109^

##### 2.1.12.6. Guanine nucleotide

**Fig. 8:**
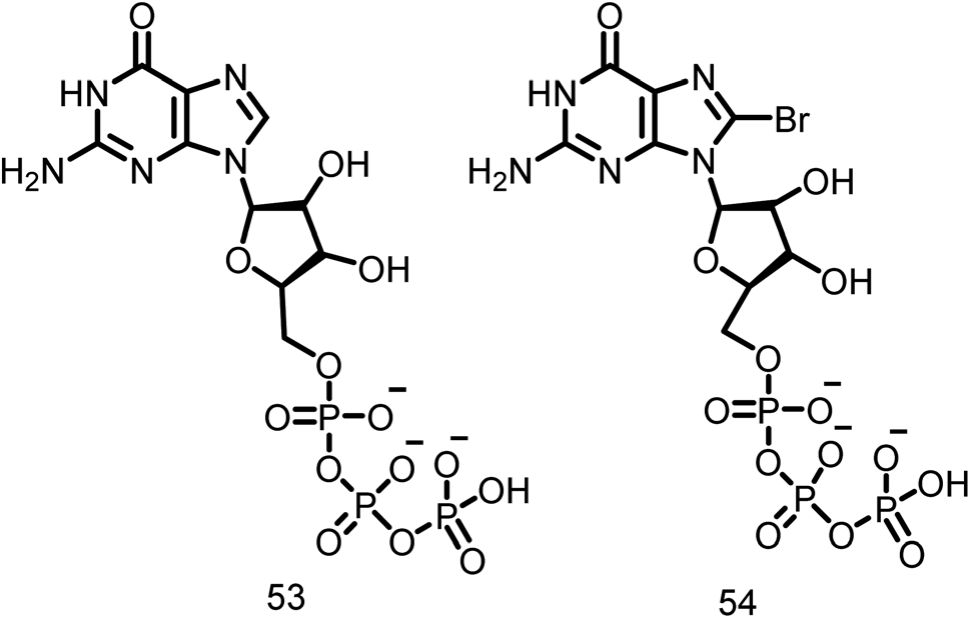
Chemical structures of guanine derivatives (53-54).

Lappchen and colleagues developed a specific inhibitor of FtsZ utilizing the structure of its natural substrate GTP (53, Fig. 8) and demonstrated the inhibitory effects of 8-bromoguanosine 5′-triphosphate (BrGTP) (54, Fig. 8). BrGTP was found to be a competitive inhibitor of both FtsZ polymerization and GTPase activity, with a *K*_i_ value of 31.8 ± 4.1 μM for GTPase activity.^110^

##### 2.1.12.7. Vanillin derivatives

**Fig. 9:**
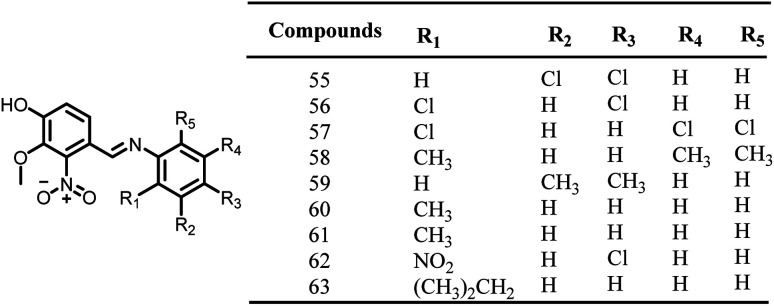
Chemical structures of vanillin derivatives (55–63).

In the year 2014, Sun and colleagues synthesized, and assessed the pharmacological activities of new vanillin derivatives as potential FtsZ inhibitors. Compound 63, Fig. 9, one of the twenty synthesized vanillin analogues, exhibited potent antibacterial activity when tested against *E. coli* (MIC: 0.28 μg ml^−1^) strains as opposed to *B. subtilis*, *P. aeruginosa*, and *S. aureus* strains, With the addition of two chlorine groups to the benzene ring, vanillin derivatives were found to have decreased antibacterial activity (31.02 to 45.67 μg ml^−1^) (55–57). In accordance with docking experiments, the compound 63 interacted with the FtsZ protein complex structure (PDB ID 2VAM) through hydrogen bonds at Asp46, Ala73, Gly108, and Arg143. Additionally, the complex was also stabilized by forming a π-cationic interaction between the benzene ring and the amino acid Arg143. Future research may take advantage of the effectiveness of vanillin analogues to create FtsZ-targeted antibacterial agents.^111^

##### 2.1.12.8. Substituted 1,6 diphenyl naphthalenes

**Fig. 10:**
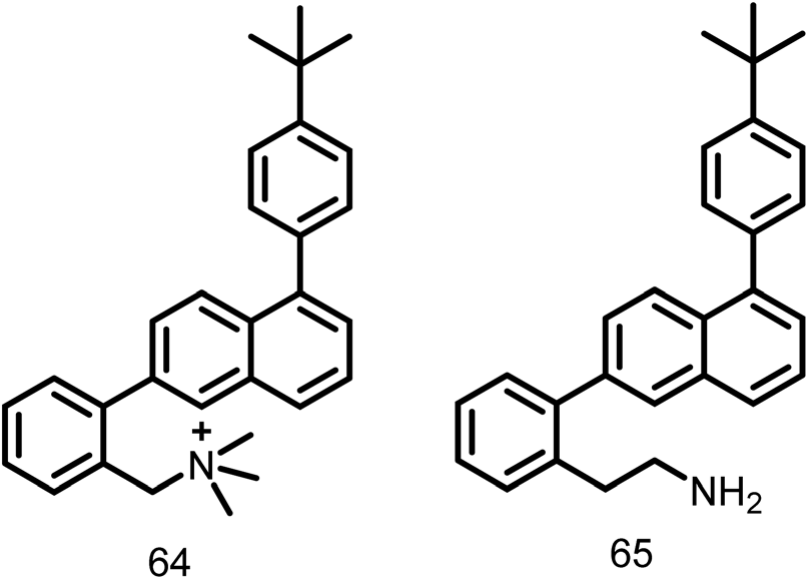
Chemical structures of substituted 1,6 diphenyl naphthalenesderivatives (64-65).

A group of substituted 1,6-diphenyl naphthalenes (DPN) were synthesized by Zhang Y. *et al.* in the year 2013. The synthesized compounds were screened for the antibacterial activity using microbroth dilution technique. The MIC values of all these substituted DPN compounds against methicillin-sensitive and resistant *S. aureus* (MSSA and MRSA) and vancomycin-sensitive and vancomycin resistant *Enterococcus faecalis* (VSE and VRE) were in the range of 0.5–64 μg ml^−1^. The compound 64 (*N*,*N*,*N*,-trimethyl ammonium derivative) (Fig. 10) exhibited a MIC value of 0.5 μg ml^−1^ (MSSA and MRSA) and 4 μg ml^−1^ (VSE and VRE). The other compound in the series, 65 (2-aminoethyl analog) (Fig. 10) showed antibacterial activity at 2 μg ml^−1^ (MSSA and MRSA) and 4 μg ml^−1^ (VSE and VRE). Authors also correlated the antibacterial activity of compounds 64 and 65 with their capacity to induce FtsZ polymerization. Although they have a significant effect on the bacterial FtsZ polymerization, they have no effect on mammalian tubulin. Therefore, DPN can act as excellent lead molecules for the synthesis of FtsZ inhibitors.^112^

##### 2.1.12.9. Isatin derivatives

**Fig. 11:**
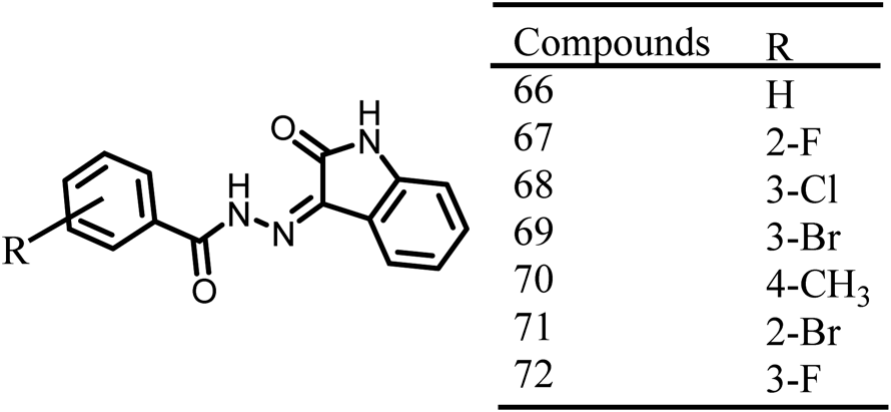
Chemical structures of isatin derivatives (66–72).

A group of isatin derivatives were created, and tested for their ability to inhibit the growth of *S. aureus*, *P. aeruginosa*, *B. subtilis*, and *E. coli* by Lian Z. M. *et al.*, 2016. Isatin compounds displayed strong antibacterial activity in comparison to vanillin derivatives. Compounds 66–72, Fig. 11, have high selectivity towards bacterial cells and among these the compounds 68 and 69 have exhibited significant antibacterial activity with IC_50_ values of 0.03 and 0.05 mmol ml^−1^ against *S. aureus*, respectively. Whereas, the compound 72 showed antibacterial activity against Gram-negative bacterial activity with IC_50_ values of 0.672 (*E. coli*) and 0.830 (*P. aeruginosa*) mmol ml^−1^.^113^

##### 2.1.12.10. Chrysophaentin

**Fig. 12:**
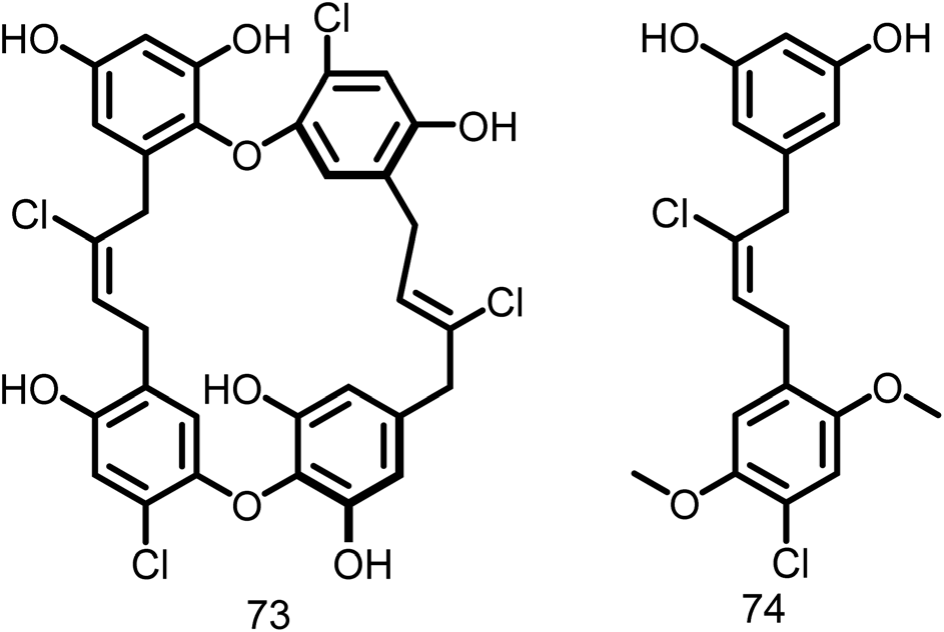
Chemical structures of chrysophaentin and hemi-chrysophaentin (73-74).

Keffer L *et al.*, 2013 introduced a novel class of natural compounds called chrysophaentins (73, Fig. 12), and screened for their antibacterial activity against drug-sensitive and drug-resistant Gram-positive bacteria. Additionally, they discovered a hemi-chrysophaentin (74, Fig. 12) with an antibacterial profile similar to that of natural products through chemical synthesis. Later they demonstrated that chrysophaentin A exhibited significant activity against the EcFtsZ (IC50: 9.9 ± 2.5 μM) and SaFtsZ GTPase (IC50: 67 ± 13 μM). Whereas hemi-chrysophaentin 2 inhibited the hydrolysis activity of EcFtsZ (IC50: 37 ± 7 μM) and SaFtsZ (IC50 of 38 ± 9 μM). Based on the results, it is clear that chrysophaentin and hemi-chrysophaentin may serve as suitable candidates for the development of FtsZ inhibitors.^114^

##### 2.1.12.11. Pyridopyrazine and pyrimidothiazine analogues

**Fig. 13:**
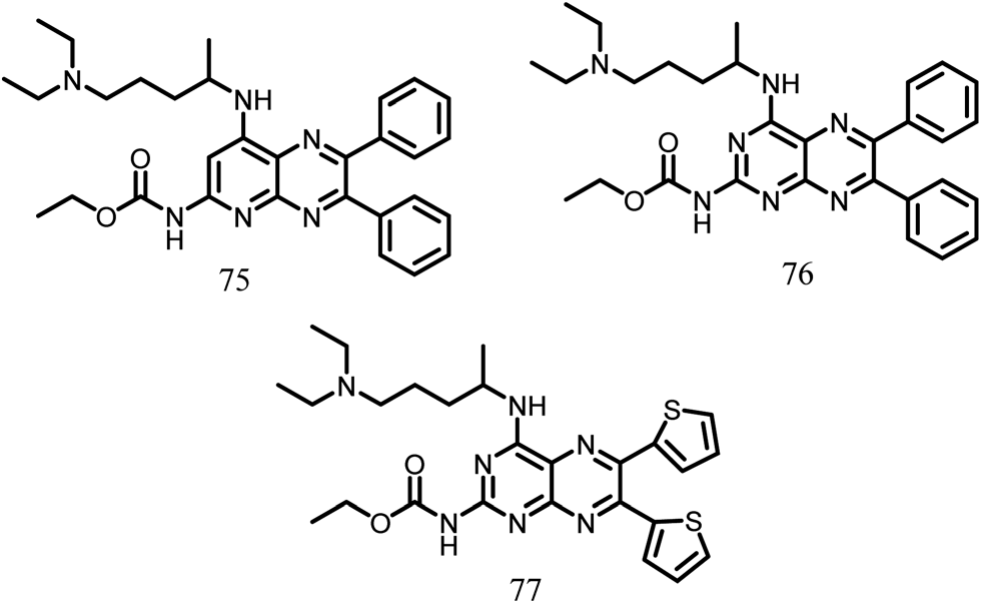
Chemical structures of pyridopyrazine and pyrimidothiazine (75–77).

Using the basic structures of the 3-deazapteridine compounds 75 and 76(Fig. 13), Mathew *et al.* in the year 2011 produced pyridopyrazine and pyrimidothiazine analogues to enhance their efficacy against FtsZ, antibacterial activity, and to lower off-target toxicity. All the synthesized compounds were tested against *M. tuberculosis* H37Ra, *M. tuberculosis* H37Rv and Vero cells. The majority of the chemicals in the molecular test inhibited FtsZ without affecting tubulin. Amongst the synthesized compounds, the compound 77 (MIC value: 0.23 μg ml^−1^), demonstrated the significant activity in comparison to the reference compound 75 (Fig. 13).^115^

##### 2.1.12.12. DAPI

**Fig. 14:**
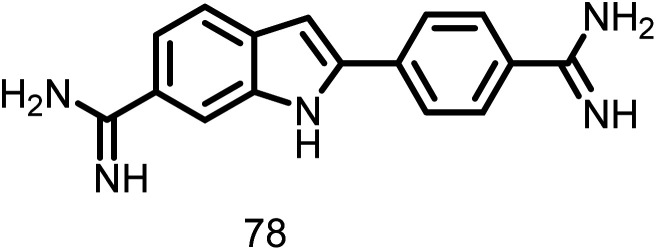
Chemical structures of 4′,6-diamidino-2-phenylindole (78).

Nova *et al.*, 2007 expanded their outstanding work on tubulin by studying 4′,6-diamidino-2-phenylindole (DAPI) (78, Fig. 14), a fluorescence probe with high binding affinity that is located on the main body (tubulin S) and shielded by the C-terminal region. Authors also characterized the interaction of DAPI with *E. coli* FtsZ. The FtsZ protofilament was shown to assimilate upon binding, which inhibited GTPase activity. The GTPase experiment demonstrated the significant antibacterial potential of DAPI with a *K*_i_ of 29.4 ± 0.3 μM and inhibited *E. coli* FtsZ noncompetitively. When DAPI was titrated, the fluorescence anisotropy was evaluated. This resulted in a dissociation constant measurement of 16.6 mM, which suggests that the protofilament was bundled and that GTPase activity was inhibited. These findings support the inhibitory effect of DAPI on *E. coli* FtsZ GTPase activity, which in turn promotes the integrity of the polymer during the polymerization process and is therefore relevant to the production of antibacterial drugs.^116^

##### 2.1.12.13. OTBA

**Fig. 15:**
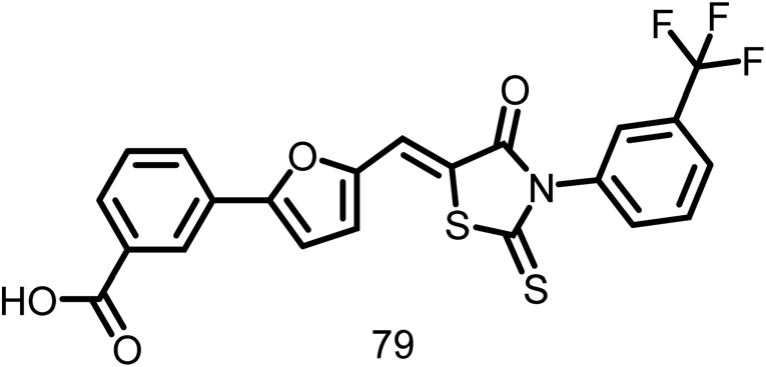
Chemical structures of OTBA (3-5-[[4-oxo-2-thioxo-3-(3-trifluoromethylphenyl)-thiazolidin-5-ylidenemethyl]-thiazolidin-5-ylidenemethyl]-furan-2-ylbenzoic acid) (79).

A rhodanine class of compound, OTBA (3-5-[[4-oxo-2-thioxo-3-(3-trifluoromethylphenyl)-thiazolidin-5-ylidenemethyl]-thiazolidin-5-ylidenemethyl]-furan-2-ylbenzoic acid), (Fig. 15), was discovered by Beuria *et al.*, in the year 2009 through screening of 81 different compounds. This compound was found to disturb the formation and function of the Z-ring by altering FtsZ assembly dynamics. The apparent dissociation constant for the binding of OTBA to FtsZ is 15 ± 1.5 μM. In the presence of 25 μM OTBA, *E. coli* FtsZ was more sensitive to light scattering by a factor of around 3, indicating that FtsZ protofilaments are more tightly bundled. Additionally, it was discovered that OTBA inhibited the growth of *B. subtilis* 168 cells with a MIC of 2 μM, demonstrating the drug's effectiveness as an antibiotic. They also contrasted OTBA's mode of action with that of the most effective anticancer drug, paclitaxel (Taxol), which inhibits the formation of the mitotic spindle by stabilizing microtubules in mammalian cells. As a result, OTBA can be recognized as an effective, selective, less hazardous FtsZ-targeting antibacterial agent.^117^

### 2.2. Antitubulin strategy

**Fig. 16:**
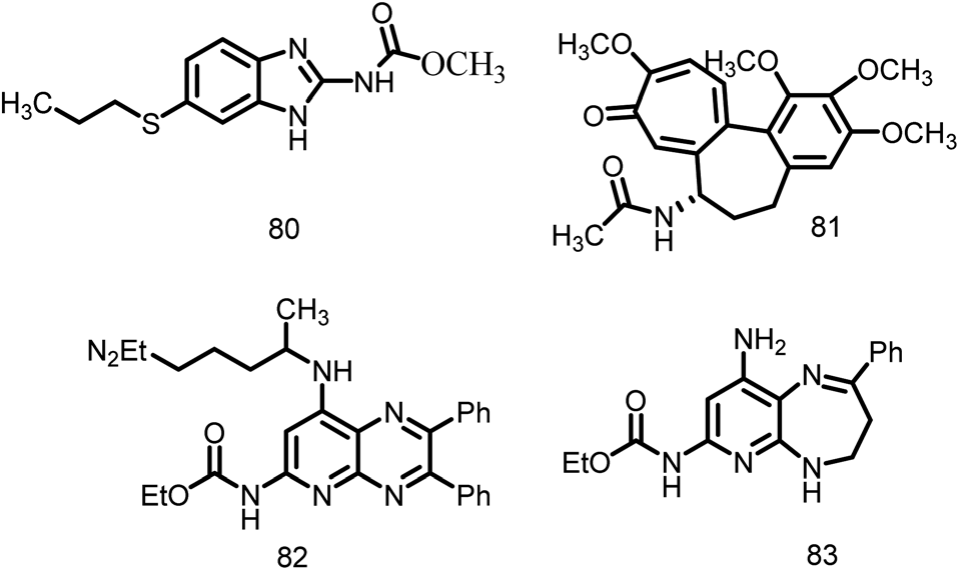
Chemical structures of tubulin inhibitors (80–83).

Finding compounds that do not target eukaryotic tubulin, sometimes known as the “antitubulin strategy,” is a problem in the search for FtsZ inhibitors. The fact that many of the traditional tubulin inhibitors are ineffective against FtsZ polymerization and GTPase activity shows that target specificity is feasible. The tubulin inhibitors 80 and 81, (Fig. 16), which both exhibit negligible effect against FtsZ polymerization and GTPase activity, are examples of this selectivity. In a similar manner, an inhibitor's cross-species activity can theoretically be tailored to specifically target FtsZ. From a synthetic tubulin inhibitor library, inhibitors 82 and 83 (Fig. 16) were found to be *M. tuberculosis* FtsZ inhibitors. A SAR analysis increased the compounds' antibacterial activity and their selectivity for inhibiting FtsZ over tubulin.^118^

## 3. Detection and quantification of FtsZ-drug complexes

The evaluation of direct drug–protein interactions is necessary for the discovery of new antibacterial agents that target FtsZ. However, the binding affinities of the discovered candidates must be measured in order to establish the minimal inhibitory concentrations (MICs). Fluorescence anisotropy is one technique that has been used for this purpose. Size changes, such as those that take place when a tiny fluorescent species binds to a big macromolecule, can drastically alter anisotropy.^119,120^ The fluorescent analogue 2′/3′ -*O*-(*N*-methylanthraniloyl) (mant)-GTP has been used in an anisotropy-based competition test to find drugs that target the GTP binding region of FtsZ. This technique has been used to examine the interactions between several synthetic inhibitors of bacterial cell division and *Bacillus subtilis* FtsZ, as well as the binding of substances like C8-substituted GTP analogues to FtsZ from the archaeon *Methanococcus jannaschii*.^108,110^

Isothermal titration calorimetry (ITC), which measures the heat change produced during contact, is a reference method for the thermodynamic characterization of binding processes.^121^ This method is therefore perfect for finding inhibitory substances and choosing candidates with the best affinity for their intended targets. As with other targets, the use of bioinformatics tools and structural techniques like nuclear magnetic resonance spectroscopy (NMR) and X-ray crystallography has contributed in the discovery and characterization of hit compounds acting on FtsZ.^29^ By docking onto the available FtsZ structures, it is possible to determine the precise binding sites of the candidates and predict chemical changes that would increase affinity or specificity. It has been carefully examined elsewhere how to determine the direct binding of inhibitors to FtsZ using techniques like NMR and X-ray crystallography.^4^

## 4. Approaches to determine the drugs targeting FtsZ polymerization

Given the critical role that GTP-induced FtsZ polymerization plays in cell division, altering this process using drugs that either inhibit assembly or hinder disassembly has been the most actively studied method currently available for affecting the function of this protein. In order to identify these substances, pinpoint their specific modes of action, and establish their optimal dosages, FtsZ polymerization measurement techniques are quite beneficial. Light scattering (LS-90°) is the most extensively used approach which can detect polymer generation, evaluate time-dependent polymer disassembly, and calculate the threshold concentration for polymerization.^122^ Kaul M. *et al.*, 2012 used this approach to investigate the inhibitory capacity of diamidino-2-phenylindole (DAPI) against FtsZ polymer dynamics.^123^ Centrifugation is other method that can be used to identify GTP-induced FtsZ polymers, followed by an assessment of the protein content of the pellet and supernatant, such as by polyacrylamide gel electrophoresis. Ruiz-Avila *et al.*, 2013 used this method to evaluate the effects of the polyhydroxy aromatic ligand UCM05 (ref. 108) and Adam D. W. *et al.*,2011 studied the influence of compound 8j, a benzamide antibiotic on the FtsZ polymerization in *Bacillus subtilis*.^124^

Dynamic light scattering (DLS) is another technique for characterizing FtsZ polymers. The random diffusive mobility of macromolecules in solution is correlated with their mass and shape and results in variations in scattered light, is what causes the autocorrelation functions found by DLS. As long as there is a mass differential of at least four times, analysis of these curves produces translational diffusion coefficients^125^ that can represent changes in mass, such as those caused by the breakdown of protein–protein interactions following drug binding. The intensity of dispersed light may be observed over time to decipher more about the polymerization/de-polymerization dynamics. DLS measurements are suitable for high throughput compound screening owing to commercial plate readers, which are available.^126^ Hou S. *et al.*, 2012 employed DLS to determine stabilizing impact of the benzamide derivative PC190723 on the FtsZ filaments of *Caulobacter crescentus*.^127^

Tunable resistive pulse sensing (TRPS) is a new technique for measuring the size of a wide range of particles (size range of 40 nm to 10 μm). It monitors the current variations caused by a species passing through a dynamically resizable pore, enabling for the simultaneous measurement of the species' size and concentration in polydisperse samples. TRPS is a very accurate and efficient technology that may be used to identify and quantify the impact of medications on the FtsZ polymerization.^128^

Fluorescence correlation spectroscopy (FCS), a single molecule method that produces autocorrelation curves from variations in fluorescence intensities, has also been used to research FtsZ polymerization.^120,129^ These parameters are used to calculate the translational diffusion of the fluorescent molecules. This method was found to be appropriate for high-throughput screening of the compounds.^130^ The suppression of FtsZ polymerization by kil peptide produced from an infecting λ phage was studied by Hernándaz-Rocamora *et al.*, 2015 using FCS and sedimentation velocity (SV) in combination, with potential use in the generation of antibiotics to treat bacterial infections.^131^

As an extension of FCS, fluorescence cross-correlation spectroscopy (FCCS) uses two-channel detection to produce cross-correlation curves that are suggestive of complex development. In order to find substances that interfere with FtsZ self-interactions, large throughput screens were conducted using FCCS by Mikuni S. *et al.*, 2015. In this test, fluorescent proteins emitting at various wavelengths were used to identify the N- and C-terminal fragments of FtsZ, and hits were determined by their capacity to lessen the cross-correlation that results from the fragment interaction with GTP.^132^ The detection of compounds that prevent FtsZ assembly may also be possible using fluorescence-based techniques like Förster resonance energy transfer (FRET) or anisotropy for the determination of the critical concentration of FtsZ polymerization.^133,134^ Chen Y. *et al.*, 2005 created a FRET-based assay for the assembly of FtsZ, altered a nontoxic surface residue to cysteine, and labelled various pools with both fluorescein (donor) and tetramethylrhodamine (acceptor). Assembly resulted in a linearly proportionate FRET signal to FtsZ concentration after the pools were combined and GTP was introduced.^133^ Further study by Reija B. *et al.*, 2011 also employed the method of fluorescence anisotropy for the identification of the minimum concentration necessary for FtsZ polymerization.^134^

The imaging techniques that offer structural information are typically used in conjunction with the biophysical and biochemical techniques mentioned above. The most common of these imaging techniques is electron microscopy. This method has been utilized to identify drug-induced FtsZ polymer bundling or structural changes in FtsZ polymers. Conventional fluorescence imaging techniques can't be used for this purpose due to their poorer resolution, which makes it impossible to characterize the small polymers that FtsZ produces in diluted solution. They may, however, be utilized to study the change of FtsZ bundles observed under crowding circumstances *in vitro* by antibacterial drugs, and they are one of the favored techniques for investigating pharmacological modes of action *in vivo*.^135^

## Conclusion

In order to combat ABR, it is urgently necessary to identify a novel antibacterial target that is crucial for bacterial survival. As a promising therapeutic target for a new class of antibacterial drugs, FtsZ is a well-researched but underutilized bacterial cytosolic protein. Availability of crystal structures of FtsZ from many bacterial species offers a plethora of information about the structure of FtsZ as well as a tremendous opportunity for structure-based drug discovery. According to their action both *in vitro* and *in vivo*, a number of FtsZ inhibitors have been described, and their target identification process rely on this information. This article primarily covers FtsZ inhibitors (peptides, natural compounds, and synthetic small molecules, particularly peptides) that can alter bacterial cytokinesis, as well as their modes of action (such as reducing FtsZ assembly, GTPase activity, and disrupting Z-ring formation). Most FtsZ inhibitors have strong antibacterial action against Gram-positive and Gram-negative bacterial strains, as well as low cytotoxicity and resistance to mammalian cells. The development of novel FtsZ inhibitors derived from natural sources would be achieved by target validation assays with sophisticated technology and effective screening procedures. Since FtsZ was a homologue of human tubulin, hence anti-tubulin strategy provided more chance for the discovery of FtsZ inhibitors. However, some recently identified FtsZ inhibitors mentioned in the review, including the natural compound viriditoxin and the synthetic compound PC190723, exhibited potent inhibition of FtsZ without any observable effects on tubulin. Positive findings from these investigations indicate that FtsZ inhibitors have potential as antibacterial agents and are quite likely to enter clinical treatment in the upcoming years. Overall, FtsZ serve as an unexplored target for developing novel antibiotics even three decades after its pivotal function in bacterial cell division was discovered.

## Author contributions

Sumaiya Kifayat: original draft writing; Vidyasrilekha Yele: original draft writing-reviewing and editing, data curation; Akram Ashames: funding acquisition, reviewing; Dilep Kumar Sigalapalli, Richie R. Bhandare, Afzal B. Shaik, and Venkatarathnam Nasipireddy: reviewing; Bharat Kumar Reddy Sanapalli: supervision, and conceptualization. All authors have read and agreed to the published version of the manuscript.

## Conflicts of interest

The authors declare no conflict of interest.

## Supplementary Material

## References

[cit1] Walsh C., Wright G. (2005). Chem. Rev..

[cit2] Silber N., Matos de Opitz C. L., Mayer C., Sass P. (2020). Future Microbiol..

[cit3] Haeusser D. P., Margolin W. (2016). Nat. Rev. Microbiol..

[cit4] Kusuma K. D., Payne M., Ung A. T., Bottomley A. L., Harry E. J. (2019). ACS Infect. Dis..

[cit5] Andreu J. M., Schaffner-Barbero C., Huecas S., Alonso D., Lopez-Rodriguez M. L., Ruiz-Avila L. B., Núñez-Ramírez R., Llorca O., Martín-Galiano A. J. (2010). J. Biol. Chem..

[cit6] Nogales E., Wolf S. G., Downing K. H. (1998). Nature.

[cit7] Mukherjee A., Dai K., Lutkenhaus J. (1993). Proc. Natl. Acad. Sci. U. S. A..

[cit8] Williamson D. L. (1974). J. Bacteriol..

[cit9] Bermudes D., Hinkle G., Margulis L. (1994). Microbiol. Rev..

[cit10] Iashnaia M. , Obydennov K., Kanwugu O., Kalinina T. and Glukhareva T., 2022

[cit11] Tiwari A. and Singh S., in Bioinformatics, Elsevier, 2022, pp. 207–217

[cit12] Lu X., Wang Y., Guo W., Zhang Z., Hu X., Nie T., Yang X., Li C., Wang X., Li X. (2023). Microbiol. Spectrum.

[cit13] Lutkenhaus J., Wolf-Watz H., Donachie W. (1980). J. Bacteriol..

[cit14] Hirota Y., Ryter A., Jacob F. (1968). Cold Spring Harbor Symp. Quant. Biol..

[cit15] Vaughan S., Wickstead B., Gull K., Addinall S. G. (2004). J. Mol. Evol..

[cit16] Kalman S., Mitchell W., Marathe R., Lammel C., Fan J., Hyman R. W., Olinger L., Grimwood J., Davis R. W., Stephens R. (1999). Nat. Genet..

[cit17] Pilhofer M., Rappl K., Eckl C., Bauer A. P., Ludwig W., Schleifer K.-H., Petroni G. (2008). J. Bacteriol..

[cit18] Stephens R. S., Kalman S., Lammel C., Fan J., Marathe R., Aravind L., Mitchell W., Olinger L., Tatusov R. L., Zhao Q. (1998). Science.

[cit19] Nakabachi A., Yamashita A., Toh H., Ishikawa H., Dunbar H. E., Moran N. A., Hattori M. (2006). Science.

[cit20] Glass J. I., Lefkowitz E. J., Glass J. S., Heiner C. R., Chen E. Y., Cassell G. H. (2000). Nature.

[cit21] Jaffe J. D., Stange-Thomann N., Smith C., DeCaprio D., Fisher S., Butler J., Calvo S., Elkins T., FitzGerald M. G., Hafez N. (2004). Genome Res..

[cit22] Adams D. W., Errington J. (2009). Nat. Rev. Microbiol..

[cit23] Tripathy S., Sahu S. K. (2019). Bioorg. Chem..

[cit24] Hamoen L. W., Meile J. C., De Jong W., Noirot P., Errington J. (2006). Mol. Microbiol..

[cit25] Weart R. B., Lee A. H., Chien A.-C., Haeusser D. P., Hill N. S., Levin P. A. (2007). Cell.

[cit26] Handler A. A., Lim J. E., Losick R. (2008). Mol. Microbiol..

[cit27] Cordell S. C., Robinson E. J., Löwe J. (2003). Proc. Natl. Acad. Sci. U. S. A..

[cit28] Haeusser D. P., Schwartz R. L., Smith A. M., Oates M. E., Levin P. A. (2004). Mol. Microbiol..

[cit29] Den Blaauwen T., Andreu J. M., Monasterio O. (2014). Bioorg. Chem..

[cit30] Lock R. L., Harry E. J. (2008). Nat. Rev. Drug Discovery.

[cit31] Alberti S., Dormann D. (2019). Annu. Rev. Genet..

[cit32] Fisher R. A., Gollan B., Helaine S. (2017). Nat. Rev. Microbiol..

[cit33] Haranahalli K., Tong S., Ojima I. (2016). Bioorg. Med. Chem..

[cit34] Carro L. (2019). Antibiotics.

[cit35] Brown D. G., Lister T., May-Dracka T. L. (2014). Bioorg. Med. Chem. Lett..

[cit36] Anand P., Thomas S. G., Kunnumakkara A. B., Sundaram C., Harikumar K. B., Sung B., Tharakan S. T., Misra K., Priyadarsini I. K., Rajasekharan K. N. (2008). Biochem. Pharmacol..

[cit37] Rai D., Singh J. K., Roy N., Panda D. (2008). Biochem. J..

[cit38] Kaur S., Modi N. H., Panda D., Roy N. (2010). Eur. J. Med. Chem..

[cit39] Domadia P., Swarup S., Bhunia A., Sivaraman J., Dasgupta D. (2007). Biochem. Pharmacol..

[cit40] Li X., Sheng J., Huang G., Ma R., Yin F., Song D., Zhao C., Ma S. (2015). Eur. J. Med. Chem..

[cit41] Kontogiorgis C., Detsi A., Hadjipavlou-Litina D. (2012). Expert Opin. Ther. Pat..

[cit42] Huecas S., Canosa-Valls A. J., Araújo-Bazán L., Ruiz F. M., Laurents D. V., Fernández-Tornero C., Andreu J. M. (2020). FEBS J..

[cit43] Heffron T. P., Heald R. A., Ndubaku C., Wei B., Augistin M., Do S., Edgar K., Eigenbrot C., Friedman L., Gancia E. (2016). J. Med. Chem..

[cit44] Ferrer-González E., Fujita J., Yoshizawa T., Nelson J. M., Pilch A. J., Hillman E., Ozawa M., Kuroda N., Al-Tameemi H. M., Boyd J. M. (2019). Sci. Rep..

[cit45] Läppchen T., Pinas V. A., Hartog A. F., Koomen G.-J., Schaffner-Barbero C., Andreu J. M., Trambaiolo D., Löwe J., Juhem A., Popov A. V. (2008). Chem. Biol..

[cit46] Ruiz F. M., Huecas S., Santos-Aledo A., Prim E. A., Andreu J. M., Fernández-Tornero C. (2022). PLoS Biol..

[cit47] Wagstaff J. M., Tsim M., Oliva M. A., García-Sanchez A., Kureisaite-Ciziene D., Andreu J. M., Löwe J. (2017). MBio.

[cit48] Huecas S., Araújo-Bazán L., Ruiz F. M., Ruiz-Ávila L. B., Martínez R. F., Escobar-Peña A., Artola M., Vázquez-Villa H., Martín-Fontecha M., Fernández-Tornero C. (2021). J. Med. Chem..

[cit49] Stokes N. R., Baker N., Bennett J. M., Chauhan P. K., Collins I., Davies D. T., Gavade M., Kumar D., Lancett P., Macdonald R. (2014). Bioorg. Med. Chem. Lett..

[cit50] Fujita J., Maeda Y., Mizohata E., Inoue T., Kaul M., Parhi A. K., LaVoie E. J., Pilch D. S., Matsumura H. (2017). ACS Chem. Biol..

[cit51] Raymond A., Lovell S., Lorimer D., Walchli J., Mixon M., Wallace E., Thompkins K., Archer K., Burgin A., Stewart L. (2009). BMC Biotech..

[cit52] Leung A. K., White E. L., Ross L. J., Reynolds R. C., DeVito J. A., Borhani D. W. (2004). J. Mol. Biol..

[cit53] Tan C. M., Therien A. G., Lu J., Lee S. H., Caron A., Gill C. J., Lebeau-Jacob C., Benton-Perdomo L., Monteiro J. M., Pereira P. M. (2012). Sci. Transl. Med..

[cit54] Alnami A., Norton R. S., Pena H. P., Haider S., Kozielski F. (2021). J. Mol. Biol..

[cit55] Matsui T., Han X., Yu J., Yao M., Tanaka I. (2014). J. Biol. Chem..

[cit56] Matsui T., Yamane J., Mogi N., Yamaguchi H., Takemoto H., Yao M., Tanaka I. (2012). Acta Crystallogr., Sect. D: Biol. Crystallogr..

[cit57] Duggirala S., Nankar R. P., Rajendran S., Doble M. (2014). Appl. Biochem. Biotechnol..

[cit58] Zang Y. (2020). Nat. Prod. Commun..

[cit59] Domadia P. N., Bhunia A., Sivaraman J., Swarup S., Dasgupta D. (2008). Biochemistry.

[cit60] Sun N., Chan F.-Y., Lu Y.-J., Neves M. A., Lui H.-K., Wang Y., Chow K.-Y., Chan K.-F., Yan S.-C., Leung Y.-C. (2014). PLoS One.

[cit61] Kim M. B., O'Brien T. E., Moore J. T., Anderson D. E., Foss M. H., Weibel D. B., Ames J. B., Shaw J. T. (2012). ACS Med. Chem. Lett..

[cit62] Bhattacharya A., Jindal B., Singh P., Datta A., Panda D. (2013). FEBS J..

[cit63] Hemaiswarya S., Soudaminikkutty R., Narasumani M. L., Doble M. (2011). J. Med. Microbiol..

[cit64] Wolff J., Knipling L. (1993). Biochemistry.

[cit65] Suzuki K., Nozawa K., Nakajima S., Kawai K.-i. (1990). Chem. Pharm. Bull..

[cit66] Wang J., Galgoci A., Kodali S., Herath K. B., Jayasuriya H., Dorso K., Vicente F., González A., Cully D., Bramhill D. (2003). J. Biol. Chem..

[cit67] Panda P., Taviti A. C., Satpati S., Kar M. M., Dixit A., Beuria T. K. (2015). Biochem. J..

[cit68] Urgaonkar S., La Pierre H. S., Meir I., Lund H., RayChaudhuri D., Shaw J. T. (2005). Org. Lett..

[cit69] Araújo-Bazán L., Ruiz-Avila L. B., Andreu D., Huecas S., Andreu J. M. (2016). Front. Microbiol..

[cit70] Ray S., Kumar A., Panda D. (2013). Biochemistry.

[cit71] Ray S., Dhaked H. P. S., Panda D. (2014). Biochemistry.

[cit72] Shimotohno K. W., Kawamura F., Natori Y., Nanamiya H., Magae J., Ogata H., Endo T., Suzuki T., Yamaki H. (2010). Biol. Pharm. Bull..

[cit73] Bi E., Lutkenhaus J. (1993). J. Bacteriol..

[cit74] Haeusser D. P., Hoashi M., Weaver A., Brown N., Pan J., Sawitzke J. A., Thomason L. C., Court D. L., Margolin W. (2014). PLos Genet..

[cit75] Paradis-Bleau C., Sanschagrin F., Levesque R. C. (2004). J. Antimicrob. Chemother..

[cit76] Van de Velde W., Zehirov G., Szatmari A., Debreczeny M., Ishihara H., Kevei Z., Farkas A., Mikulass K., Nagy A., Tiricz H. (2010). Science.

[cit77] Farkas A., Maróti G., Dürgő H., Györgypál Z., Lima R. M., Medzihradszky K. F., Kereszt A., Mergaert P., Kondorosi É. (2014). Proc. Natl. Acad. Sci. U. S. A..

[cit78] Sass P., Josten M., Famulla K., Schiffer G., Sahl H.-G., Hamoen L., Brötz-Oesterhelt H. (2011). Proc. Natl. Acad. Sci. U. S. A..

[cit79] Clement M.-J., Jourdain I., Lachkar S., Savarin P., Gigant B., Knossow M., Toma F., Sobel A., Curmi P. A. (2005). Biochemistry.

[cit80] Clement M.-J., Kuoch B.-t., Ha-Duong T., Joshi V., Hamon L., Toma F., Curmi P. A., Savarin P. (2009). Biochemistry.

[cit81] Tiricz H., Szűcs A., Farkas A., Pap B., Lima R. M., Maróti G., Kondorosi É., Kereszt A. (2013). Appl. Environ. Microbiol..

[cit82] Ghosal A., Vitali A., Stach J. E., Nielsen P. E. (2013). ACS Chem. Biol..

[cit83] Good L., Awasthi S. K., Dryselius R., Larsson O., Nielsen P. E. (2001). Nat. Biotechnol..

[cit84] Liang S., He Y., Xia Y., Wang H., Wang L., Gao R., Zhang M. (2015). Int. J. Infect. Dis..

[cit85] Somma A. D., Avitabile C., Cirillo A., Moretta A., Merlino A., Paduano L., Duilio A., Romanelli A. (2020). Biochim. Biophys. Acta, Gen. Subj..

[cit86] Somma A. D., Canè C., Moretta A., Duilio A. (2021). Antibiotics.

[cit87] Magiorakos A.-P., Srinivasan A., Carey R. B., Carmeli Y., Falagas M., Giske C., Harbarth S., Hindler J., Kahlmeter G., Olsson-Liljequist B. (2012). Clin. Microbiol. Infect..

[cit88] Lazzaro B. P., Zasloff M., Rolff J. (2020). Science.

[cit89] Molchanova N., Hansen P. R., Franzyk H. (2017). Molecules.

[cit90] Bisson-Filho A. W., Discola K. F., Castellen P., Blasios V., Martins A., Sforça M. L., Garcia W., Zeri A. C. M., Erickson H. P., Dessen A. (2015). Proc. Natl. Acad. Sci. U. S. A..

[cit91] Corrales-Guerrero L., Steinchen W., Ramm B., Mücksch J., Rosum J., Refes Y., Heimerl T., Bange G., Schwille P., Thanbichler M. (2022). Proc. Natl. Acad. Sci. U. S. A..

[cit92] Chen Y., Milam S. L., Erickson H. P. (2012). Biochemistry.

[cit93] Dhanoa G. K., Kushnir I., Qimron U., Roper D. I., Sagona A. P. (2022). Front. Cell. Infect. Microbiol..

[cit94] Westman E. L., Yan M., Waglechner N., Koteva K., Wright G. D. (2013). Chem. Biol..

[cit95] Cheung R. C. F., Ng T. B., Wong J. H. (2015). Mar. Drugs.

[cit96] Yang N., Liu X., Teng D., Li Z., Wang X., Mao R., Wang X., Hao Y., Wang J. (2017). Sci. Rep..

[cit97] Haag A. F., Baloban M., Sani M., Kerscher B., Pierre O., Farkas A., Longhi R., Boncompagni E., Hérouart D., Dall'Angelo S. (2011). PLoS Biol..

[cit98] Good L., Nielsen P. E. (1998). Nat. Biotechnol..

[cit99] Barkowsky G., Lemster A.-L., Pappesch R., Jacob A., Krüger S., Schröder A., Kreikemeyer B., Patenge N. (2019). Mol. Ther. – Nucleic Acids.

[cit100] Narenji H., Teymournejad O., Rezaee M. A., Taghizadeh S., Mehramuz B., Aghazadeh M., Asgharzadeh M., Madhi M., Gholizadeh P., Ganbarov K. (2020). Microb. Pathogen..

[cit101] Nekhotiaeva N., Awasthi S. K., Nielsen P. E., Good L. (2004). Mol. Ther..

[cit102] Eriksson M., Nielsen P. E., Good L. (2002). J. Biol. Chem..

[cit103] Ghosal A., Nielsen P. E. (2012). Nucleic Acid Ther..

[cit104] Simmaco M., Mignogna G., Canofeni S., Miele R., Mangoni M. L., Barra D. (1996). Eur. J. Biochem..

[cit105] Margalit D. N., Romberg L., Mets R. B., Hebert A. M., Mitchison T. J., Kirschner M. W., RayChaudhuri D. (2004). Proc. Natl. Acad. Sci. U. S. A..

[cit106] Kumar K., Awasthi D., Lee S.-Y., Zanardi I., Ruzsicska B., Knudson S., Tonge P. J., Slayden R. A., Ojima I. (2011). J. Med. Chem..

[cit107] Ray S., Jindal B., Kunal K., Surolia A., Panda D. (2015). FEBS J..

[cit108] Ruiz-Avila L. B., Huecas S., Artola M., Vergoñós A., Ramírez-Aportela E., Cercenado E., Barasoain I., Vázquez-Villa H., Martín-Fontecha M., Chacón P. (2013). ACS Chem. Biol..

[cit109] Huang Q., Kirikae F., Kirikae T., Pepe A., Amin A., Respicio L., Slayden R. A., Tonge P. J., Ojima I. (2006). J. Med. Chem..

[cit110] Huecas S., Schaffner-Barbero C., García W., Yébenes H., Palacios J. M., Díaz J. F., Menéndez M., Andreu J. M. (2007). J. Biol. Chem..

[cit111] Sun J., Li M.-H., Wang X.-Y., Zhang Y., Yuan R.-J., Liu H.-Y., Zhu H.-L. (2014). Med. Chem. Res..

[cit112] Zhang Y., Giurleo D., Parhi A., Kaul M., Pilch D. S., LaVoie E. J. (2013). Bioorg. Med. Chem. Lett..

[cit113] Lian Z.-M., Sun J., Zhu H.-L. (2016). J. Mol. Struct..

[cit114] Keffer J. L., Huecas S., Hammill J. T., Wipf P., Andreu J. M., Bewley C. A. (2013). Bioorg. Med. Chem..

[cit115] Mathew B., Srivastava S., Ross L. J., Suling W. J., White E. L., Woolhiser L. K., Lenaerts A. J., Reynolds R. C. (2011). Bioorg. Med. Chem..

[cit116] Nova E., Montecinos F., Brunet J. E., Lagos R., Monasterio O. (2007). Arch. Biochem. Biophys..

[cit117] Beuria T. K., Singh P., Surolia A., Panda D. (2009). Biochem. J..

[cit118] White E. L., Suling W. J., Ross L. J., Seitz L. E., Reynolds R. C. (2002). J. Antimicrob. Chemother..

[cit119] Jameson D. M. and Mocz G., in Protein-Ligand Interactions, Springer, 2005, pp. 301–322 10.1385/1-59259-912-5:301 15940004

[cit120] Royer C. A., Scarlata S. F. (2008). Methods Enzymol..

[cit121] Freyer M. W., Lewis E. A. (2008). Methods Cell Biol..

[cit122] Mukherjee A., Lutkenhaus J. (1999). J. Bacteriol..

[cit123] Kaul M., Parhi A. K., Zhang Y., LaVoie E. J., Tuske S., Arnold E., Kerrigan J. E., Pilch D. S. (2012). J. Med. Chem..

[cit124] Adams D. W., Wu L. J., Czaplewski L. G., Errington J. (2011). Mol. Microbiol..

[cit125] Schmitz K. , An introduction to dynamic light scattering by macromolecules, Academic Press, New York, 1990

[cit126] Hanlon A. D., Larkin M. I., Reddick R. M. (2010). Biophys. J..

[cit127] Hou S., Wieczorek S. A., Kaminski T. S., Ziebacz N., Tabaka M., Sorto N. A., Foss M. H., Shaw J. T., Thanbichler M., Weibel D. B. (2012). J. Biol. Chem..

[cit128] Gross-Rother J., Blech M., Preis E., Bakowsky U., Garidel P. (2020). Pharmaceutics.

[cit129] Kitamura A., Kinjo M. (2018). Int. J. Mol. Sci..

[cit130] Wachsmuth M., Conrad C., Bulkescher J., Koch B., Mahen R., Isokane M., Pepperkok R., Ellenberg J. (2015). Nat. Biotechnol..

[cit131] Hernández-Rocamora V. M., Alfonso C., Margolin W., Zorrilla S., Rivas G. (2015). J. Biol. Chem..

[cit132] Mikuni S., Kodama K., Sasaki A., Kohira N., Maki H., Munetomo M., Maenaka K., Kinjo M. (2015). PLoS One.

[cit133] Chen Y., Erickson H. P. (2005). J. Biol. Chem..

[cit134] Reija B., Monterroso B., Jiménez M., Vicente M., Rivas G., Zorrilla S. (2011). Anal. Biochem..

[cit135] González J. M., Jiménez M., Vélez M., Mingorance J., Andreu J. M., Vicente M., Rivas G. (2003). J. Biol. Chem..

